# Effects of multidomain environmental and mental health factors on the development of empathetic behaviors and emotions in adolescence

**DOI:** 10.1371/journal.pone.0293473

**Published:** 2023-11-22

**Authors:** Calli Smith, Catherine Stamoulis

**Affiliations:** 1 Department of Pediatrics, Division of Adolescent Medicine, Boston Children’s Hospital, Boston, MA, United States of America; 2 Department of Pediatrics, Harvard Medical School, Boston, MA, United States of America; Keele University School of Medicine, UNITED KINGDOM

## Abstract

Empathy is at the core of our social world, yet multidomain factors that affect its development in socially sensitive periods, such as adolescence, are incompletely understood. To address this gap, this study investigated associations between social, environmental and mental health factors, and their temporal changes, on adolescent empathetic behaviors/emotions and, for comparison, callous unemotional (CU) traits and behaviors, in the early longitudinal Adolescent Brain Cognitive Development sample (baseline: n = 11062; 2-year follow-up: n = 9832, median age = 119 and 144 months, respectively). Caregiver affection towards the youth, liking school, having a close friend, and importance of religious beliefs/spirituality in the youth’s life were consistently positively correlated with empathetic behaviors/emotions across assessments (p<0.001, Cohen’s f = ~0.10). Positive family dynamics and cohesion, living in a neighborhood that shared the family’s values, but also parent history of substance use and (aggregated) internalizing problems were additionally positively associated with one or more empathetic behaviors at follow-up (p<0.001, f = ~0.10). In contrast, externalizing problems, anxiety, depression, fear of social situations, and being withdrawn were negatively associated with empathetic behaviors and positively associated with CU traits and behaviors (p<0.001, f = ~0.1–0.44). The latter were also correlated with being cyberbullied and/or discriminated against, anhedonia, and impulsivity, and their interactions with externalizing and internalizing issues. Significant positive temporal correlations of behaviors at the two assessments indicated positive (early) developmental empathetic behavior trajectories, and negative CU traits’ trajectories. Negative changes in mental health adversely moderated positive trajectories and facilitated negative ones. These findings highlight that adolescent empathetic behaviors/emotions are positively related to multidomain protective social environmental factors, but simultaneously adversely associated with risk factors in the same domains, as well as bully victimization, discrimination, and mental health problems. Risk factors instead facilitate the development of CU traits and behaviors.

## 1. Introduction

Empathy is a core aspect of human interactions and social behaviors [1, 2], and its development is profoundly impacted by experiential and environmental factors during the first two decades of life. Distinctions between different aspects of empathy have been debated [3–7], but there is overall agreement that its primary components are affective (sharing others’ emotions/feelings and attitudes) and cognitive (processing and interpreting others’ emotions/feelings) [8–19]. These components may be supported by distinct networks of interconnected brain regions [20–24] that undergo significant reorganization during development, particularly in periods of heightened maturation such as adolescence. In some settings, one aspect of empathy may dominate over the other (for example, cognitive over affective empathy in healthcare professionals [25], but both drive prosocial and cooperative behaviors [1, 19, 26–28]. Beyond these components, empathic motivation and compassionate empathy are reflected in behaviors and actions in response to another person’s emotional state, and are likely supported by overlapping neural circuits [12, 17, 29]. Finally, prior studies also suggest that different aspects of empathy may be interrelated as parts of a broader construct [30–33].

The development of empathy and role of protective and risk factors on its age trajectory are only partially understood. Given its central role in social behaviors and functioning groups (from familities to societies), and associations between lack of empathy and moral disengagement and aggressive, antisocial, and immoral behaviors [16, 19, 34–39], there is a significant unmet need to better understand genetic, environmental and experiential factors that contribute to its development and variability among individuals [14, 21, 31, 40–50]. In particular, it is important to unravel these factors’ contributions in adolescence, a biologically and socially vulnerable period of significant cognitive, emotional and social maturation. During adolescence, co-occurring environmental changes and unique experiences (alongside biological development) may play a critical role in shaping an individual’s social identity and prosocial behaviors, and the neural circuitry that supports them. To date, the combinatorial and time-varying effects of parent-youth relationships, parenting, parental beliefs and behaviors, family dynamics, school and teachers, peer relationships, culture, religiosity and/or spirituality, and neighborhood/community on the development of empathy in adolescence have not been systematically examined. In the context of the ecological systems theory [51] protective factors associated with parents, family, peers, and school/teachers are part of the child’s microsystem (the immediate and most impactful aspect of the youth environment) and contribute to an overall positive youth development, including prosocial and specifically empathetic behaviors. In the same context, protective factors associated with community and neighborhood are additional ecological assets, albeit at a different level of the youth environment (the exosystem). Factors associated with culture represent the youth’s ecological macrosystem, although religiosity/spirituality may be considered part of both the youth microsystem (assuming they are tied to parental beliefs and behaviors) and their macrosystem of ecolological influences. Across systems, all these factors interact with each other, and together influence youth social development and behaviors. It is, thus, critical to study their combinatorial, rather than individual impacts as systems on the development of empathy.

### 1.1. Environmental factors and their impact on the development of empathy

Parental/family beliefs and behaviors provide a foundation for youth empathy. Parental expressions of love and warmth may lead to more prosocial youth behaviors [52], while lack of positive expressions toward the child and rejecting parenting have been associated with lower affective and cognitive empathy, non-empathetic behaviors, and more antisocial behaviors [37, 38]. Parenting that restricts youth decisions and independence may lead to lower affective empathy, while parenting that supports and promotes youth self-expression and independence may be associated with higher empathy [53]. Warm and low-conflict relationships with siblings have been associated with increased empathy in late childhood and adolescence [54]. Thus, an overall supportive and nurturing family environment positively impacts the development of empathy [55]. Although important across the lifespan, the influence of parent behaviors and family environment on youth is likely to change throughout development—particularly across adolescence. However, even as the youth social environment expands, relationships with others beyond the family also depend on youth social behaviors that continue to be shaped by the family, i.e., bidirectional associations between individuals and social contexts [56, 57].

As the youth social world expands with age, peer relationships progressively become central to the development of prosocial behaviors. Their effects may become even stronger than those of parent-youth relationships, partly as a result of social reorientation [57]. High friendship quality in adolescents has been linked to increased empathetic responses and perspective taking, although the association may depend on friend selection [58, 59]. In contrast, negative peer interactions, such as bullying behaviors, have been associated with lower affective empathy, and being a victim of bullying has been linked to lower cognitive empathy [60, 61].

Beyond family and peers, youth spend a substantial part of their day at school, interacting with teachers, who are also part of their immediate social environment. Warm and reciprocal relationships with them are ecological assets that have been associated with increased empathy and prosocial behaviors [42, 62]. These relationships may also be mediated by student attitude towards school (for example, interesting vs boring) [63] and positive perception of school culture [62, 64]. In addition, community and neighborhood factors may positively or negatively impact the development of empathy directly or through their effects on the youth’s immediate environment. The relationship is likely bidirectional, given that individual/family socioeconomic factors correlate with community and neighborhood quality factors. In prior work, neighborhood impoverishment and violence have been associated with antisocial behaviors [37, 65, 66] and callous-unemotional (CU) traits [67].

Culture, a macroscale system aspect of the youth environment, can also impact empathy directly and indirectly, i.e., through its impact on youth but also parents, family, and peers. Feeling culturally similar to someone in distress has been associated with increased perspective taking and empathic concern [68–70]. Cultural differences may also be reflected in differences in affective empathy [71] and empathic responses [72]. A large study of over 100,000 adults across more than 60 countries reported variable levels of empathic concern in different cultures and a positive correlation between empathy and prosocial behaviors, self-esteem, well-being, and collectivism [73]. High familism has been linked to increased ethnic socialization and prosocial behaviors over time, which may, in turn, be linked to higher empathy [74, 75].

Religiosity and spirituality can also influence empathy directly or through other domains, e.g., parent and family behaviors, peer relationships, and community. Recent work based on a large cohort of pre/early adolescents reported associations between parental religiosity and changes in youth brain circuits that overlap with those supporting empathy [76]. Non-religious beliefs on spirituality but not overall religiosity have been positively associated with empathy and altruism [77], though some aspects of empathy have also been linked to being religious [78]. Studies in adolescents have reported direct and indirect positive correlations between empathy and the importance of religion or religious commitment (but not religious involvement) [79, 80]. In older adolescents and adults, spirituality has been associated with increased empathy [81, 82]. These variable findings suggest that correlations between religiosity, spirituality, and empathy may be context-dependent.

### 1.2. Mental health and its impact on the development of empathy

Empathy is also bidirectionally correlated with mental health and temperament. For example, lack of empathy is a key component of CU traits [14], which have been linked to lower cognitive and affective empathy and prosociality [83]. Anxiety may moderate these relationships [84, 85]. Multiple dimensions of anxiety also adversely impact affective and/or cognitive empathy [86, 87]. Depression has been positively associated with affective empathy but negatively associated with cognitive empathy, with moderations by self-esteem, feeling in control of one’s life, education, guilt, and executive function [88–93]. More broadly, internalizing behaviors have been associated with increased empathy and lower CU traits, and externalizing behaviors with reduced empathy and higher CU traits [39, 94, 95]. Reduced executive control, increased negative affect (anger, fear), and reduced affiliation have been associated with increased externalizing behaviors [96], which may adversely impact empathy and vice versa. Similarly, increased fear and lower security of attachment have been linked to reduced empathy, while agreeableness and conscientiousness have been linked to increased affective and cognitive empathy [97, 98].

### 1.3 Gaps in knowledge, study hypotheses and goals

Although a number of studies have reported correlations between domain-specific factors and empathy, to the best of our knowledge, no large-scale studies have examined the combined effects of multidomain environmental and mental health factors on prosocial (including empathetic) behaviors specifically in adolescence. This complex developmental period is critical to the development of social skills [99, 100], and environmental factors may significantly affect the development of empathy in ways that are difficult to disentangle and quantify, and thus need to be studied together. This is partly due to the heterogeneity of adolescent behaviors and empathy, but also the uniqueness of youth experiences and environment.

The historically large, longitudinal ABCD study [101], which follows ~12,000 children from pre/early adolescence to young adulthood, measures multiple environmental domains and multidimensional (including prosocial) behaviors. Specifically, extensive survey data on family environment, parental beliefs and attitudes, religiosity, peer relationships, school, neighborhood/community, and culture, provide a unique opportunity to study the relationship between environmental factors and empathetic behaviors in adolescence. Prior work based on the ABCD sample has focused primarily on CU traits, which are highly correlated with lack of empathy [95, 102–104]. However, comprehensive investigations of multidomain factors that may individually and combinatorially directly or indirectly affect the development of empathetic behaviors are lacking even in this cohort.

To address this significant gap in knowledge and gain a comprehensive understanding of the relative contribution of multidomain environmental factors to empathetic behaviors, the present study investigated early longitudinal data from the ABCD study (at baseline and two-year follow-up). It systematically and comprehensively assessed direct and indirect correlations between multiple environmental domains and empathetic behaviors and emotions (and, for comparison, CU traits and behaviors). These domains included family (dynamics, parental beliefs and attitudes toward the youth), school, peers, culture, religion, and neighborhood/community. Moderating relationships and interactions between factors were also assessed.

The study hypothesized that, taken together, multiple aspects of the youth social environment differentially contribute to youth empathetic behaviors, and the latter’s associations with environmental factors change as a function of development. Specifically, these empathetic behaviors are a) positively associated with cohesive family dynamics and youth-parent interactions, strong peer relationships, and positive school, community, and/or neighborhood environments, and are adversely affected by negative family dynamics, rigid parenting, and negative peer relationships, as well as risk factors in the youth’s neighborhood and/or community; b) positive and negative peer relationships and the neighborhood/community environment play an increasingly important role in the development of empathy (and/or CU traits and behaviors) as a function of age. The study also hypothesized that, in addition to direct relationships and additive effects of individual domains, cross-domain interactions also correlate with empathetic behaviors and emotions.

## 2. Methods

Deidentified publicly available human data were analyzed in this study, and are available through the National Institute of Mental Health’s repository (nda.nih.gov). The study was approved by the Institutional Review Board at Boston Children’s Hospital. All analyses were performed in accordance with relevant guidelines and regulations.

### 2a. Participants

#### 2a-1. Demographic information

This study analyzed multimodal data from the ABCD data, collected at study entry (baseline) and the 2-year follow up, at which full survey datasets (across domains of interest) were available. These data were primarily from release 4.0 (with a few exceptions, where relevant data were available in release 3.0 but not 4.0—see S1 Table in S1 File), and are available through the National Institute of Mental Health Data Archive [105]. Participants with diagnosed Autism Spectrum Disorder, bipolar disorder, Post-Traumatic Stress Disorder (PTSD), or unspecified schizophrenia spectrum or other psychotic disorders were excluded from the study, as individuals with such disorders may have significant disorder-related differences in empathy or expression of empathy and related processes, which were beyond the scope of this study [106–115]. A total of 11062 youth at baseline and 9832 who also had 2-year follow up data were studied. Based on these sample sizes, both the baseline and follow-up samples had ≥80% statistical power to detect even small effects (Cohen’s f ≥ 0.1) of 40-~50 parameters (depending on the outcome) in statistical models. The ABCD cohort demographics at study entry have been previously reported [116], and the present study’s demographics were similar. The baseline and follow-up cohorts included 5673 (51.28%) boys and 5389 (48.72%) girls, and 5089 (51.76%) boys and 4743 (48.24%) girls, respectively. Median age was 119 months at baseline (Interquartile Range (IQR) = 14, range 107–133 months), and 144 months at follow-up (IQR = 13, range 127–168 months).

The sample was primarily white (~65%) and non-Hispanic (~80%), with similar distributions at both assessments (Black ~15%, Biracial ~10%, Hispanic ~ 20%). Given the unbalanced distributions of race in this sample, race was represented in models as a dichotomous variable (white = 1 vs nonwhite = 0). Inclusion of more specific racial categories as indicator variables prevented most statistical models from converging. Pubertal information was obtained from Youth Physical Health survey and was represented by an ordinal variable (pre-puberty = 1 to post-puberty = 5). At baseline, almost 25% of participants were in pre-puberty, over 30% in early puberty, and ~25% were in mid-puberty, with the remaining participants in later pubertal stages (~20% were missing pubertal stage information). At follow-up, less than 15% were in pre-puberty, about 25% were in early puberty, almost 40% in mid puberty and about 20% in late (~5% were missing this information). Based on the Youth Acculturation Survey Modified from PhenX, almost 75% of participants at baseline and ~72% at follow-up had excellent English fluency (based on a Likert scale from Poor (= 1) to Excellent (= 4)).

#### 2a-2. Geographic information

Based on the Residential History Derived Scores (census tract data), median population density at participants’ residential addresses was 1658 people/km^2^ at baseline [IQR = 1999 people/km^2^; 611 (5.52%) were missing these data], indicating that on average participants lived in urban areas (typically with ≥ 618 people/km^2^) [117, 118]. In addition, 1855 (16.77%) lived in the Northeast, 2253 (20.37%) in the Midwest, 3113 (28.14%) in the South, and 3841 (34.72%) in the West. At follow-up, median population density was 1567 people per km^2^ [IQR = 1936 people/km^2^; 6524 (66.35%) missing data]. Of participants, 1595 (16.22%) lived in the Northeast, 1970 (20.04%) in the Midwest, 2805 (28.53%) in the South, and 3462 (35.21%) in the West. Individual geographic divisions and regions were represented by indicator variables in statistical models. Note that population density was not included in statistical models for follow-up, given that almost 70% of participants were missing these data.

At baseline, ~25% of participant families had annual combined income of <$50,000, 30% between $50,000 and $99,999, ~30% between $100,000 and $199,999, and slightly over 10% had income > $200,000 [944 (8.54%) missing data]. A similar distribution of income levels was estimated at follow-up. At baseline, primary caregivers had on average at least a Bachelor’s degree, ~30% had some college or an associate degree, and over 25% had an advanced degree [15 (0.14%) missing these data]. A similar distribution of caregiver highest education level was estimated at follow-up. Family income and caregiver education were represented by discrete (ordinal) variables in statistical models. Furthermore, both at baseline and follow-up ~75% of primary caregiver respondents were married or living with a partner. Marital status was represented by a dichotomous variable [married or living with a partner (= 1) vs other (= 0)]. Finally, almost 90% of primary caregivers at baseline and follow-up had excellent English fluency. Family and parent demographics are summarized in Table 1.

**Table 1:** Demographic data, including geographic/residential information.

		Baseline	Year 2 Follow Up
**N**		11062	9832
**Age (mos)**	Median (IQR)	119 (14)	144 (13)
	Range	107–133	127–168
	Missing	0	0
**Sex**	Girls	5389 (48.72%)	4743 (48.24%)
	Boys	5673 (51.28%)	5089 (51.76%)
	Missing	0	0
**Pubertal Stage**	Pre-puberty	2538 (22.95%)	1247 (12.68%)
	Early puberty	3455 (31.23%)	2498 (25.41%)
	Mid puberty	2712 (24.52%)	3636 (36.98%)
	Late puberty	206 (1.86%)	1846 (18.78%)
	Post puberty	19 (0.17%)	82 (0.83%)
	Missing	2132 (19.27%)	523 (5.32%)
**Race**	White	7091 (64.10%)	6423 (65.33%)
	Black/African American	1675 (15.14%)	1413 (14.37%)
	American Indian/Native American, Alaska Native	59 (0.53%)	47 (0.48%)
	Native Hawaiian, Guamanian, Samoan, Other Pacific Islander	15 (0.14%)	14 (0.14%)
	Asian Indian, Chinese, Filipino, Japanese, Korean, Vietnamese, Other Asian	235 (2.13%)	206 (2.10%)
	Biracial	1139 (10.30%)	992 (10.09%)
	Other Race (including >2 racial groups)	683 (6.17%)	597 (6.07%)
	Refuse to answer/don’t know	143 (1.29%)	118 (1.20%)
	Missing	22 (0.20%)	22 (0.22%)
**Ethnicity**	Hispanic	2246 (20.30%)	1975 (20.09%)
	Non-Hispanic	8676 (78.43%)	7746 (78.78%)
	Missing	140 (1.27%)	111 (1.13%)
**Youth English Fluency**	Poor	50 (0.45%)	16 (0.16%)
	Fair	198 (1.79%)	189 (1.92%)
	Good	2639 (23.86%)	2527 (25.70%)
	Excellent	8158 (73.75%)	7070 (71.91%)
	Missing	17 (0.15%)	30 (0.31%)
**Parent English Fluency**	Poor	233 (2.11%)	183 (1.86%)
	Fair	324 (2.93%)	293 (2.98%)
	Good	746 (6.74%)	644 (6.55%)
	Excellent	9691 (87.61%)	8561 (87.07%)
	Missing	68 (0.61%)	151 (1.54%)
**Family Income**	<5,000	359 (3.24%)	269 (2.74%)
	5,000–24,999	1053 (9.52%)	771 (7.84%)
	25,000–49,999	1451 (13.12%)	1117 (11.36%)
	50,000–99,999	2896 (26.18%)	2442 (24.84%)
	100,000–199,999	3160 (28.57%)	3088 (31.41%)
	> = 200,000	1199 (10.84%)	1320 (13.42%)
	Missing	944 (8.53%)	825 (8.39%)
**Primary Caregiver Education**	Advanced degree (Master’s professional (MD, JD, etc.) and doctoral degrees)	2870 (25.95%)	2665 (27.11%)
	Bachelor’s degree	3145 (28.43%)	2829 (28.77%)
	Associate degree	1402 (12.67%)	1249 (12.70%)
	Some College	1779 (16.08%)	1485 (15.10%)
	High School/GED	1141 (10.31%)	991 (10.08%)
	Did Not Graduate High school	710 (6.42%)	551 (5.61%)
	Missing	15 (0.14%)	62 (0.63%)
**Parent Marital Status**	Married or living with partner	8207 (74.20%)	7329 (74.54%)
	Not Married or living with partner	2767 (25.01%)	2390 (24.31%)
	Missing	88 (0.79%)	113 (1.15%)
**Census Region**	Northeast	1855 (16.77%)	1595 (16.22%)
	Midwest	2253 (20.37%)	1970 (20.04%)
	South	3113 (28.14%)	2805 (28.53%)
	West	3841 (34.72%)	3462 (35.21%)
**Census Division**	New England	1112 (10.05%)	1052 (10.70%)
	Middle Atlantic	743 (6.72%)	543 (5.52%)
	East North Central	1028 (9.30%)	886 (9.01%)
	West North Central	1225 (11.07%)	1084 (11.03%)
	South Atlantic	2420 (21.88%)	2123 (21.59%)
	East South Central	0	0
	West South Central	693 (6.26%)	682 (6.94%)
	Mountain	1486 (13.43%)	1394 (14.18%)
	Pacific	2355 (21.29%	2068 (21.03%)
**Primary Address Population Density (persons per km** ^**2**^ **)**	Median (IQR)	1658.148 (1999.172)	1566.650 (1935.949)
	Range	0–64718.640	0–60283.320
	Missing	611 (5.52%)	6524 (66.35%)

*The ‘other’ racial category includes those who reported ‘other race’ or selected more than 2 racial groups in the ABCD study.

### 2b. Multidomain environmental and other participant data

Data, primarily from parent reports (aside from youth reports on parental monitoring and caregiver warmth), were analyzed. In some environmental domains, youth survey data were not available at all at baseline. In others, responses were missing for a substantial number of participants. In these cases, if dyad surveys were available, the parent one was used. Otherwise these data were not included. Across surveys, individual questions were selected based on previously reported links to empathy: family dynamics (and parent history of substance and mental health issues), parental beliefs/values, parent attitudes/behaviors towards the child, religious and cultural background, school, neighborhood and community, and peer relationships. Youth mental health information (focusing on anxiety, social anxiety disorder, fear of social situations, depression, anhedonia, self esteem, and externalizing and internalizing behaviors) and temperament (impulsivity, being withdrawn, preferring to be alone, self conscious/easily embarrassed) were also extracted. Finally, information on sleep duration, Body Mass Index (BMI; estimated from height and weight), physical activity, and screen time was also extracted. All questions and data analyzed are summarized in S1 Table of S1 File.

#### 2b-1. Family dynamics, parental beliefs, values, and behaviors, and religious and cultural background

Questions related to the strength of parental beliefs on family and religion were extracted from the Parent Mexican American Cultural Values Scale [Likert scale, from not at all (= 1) to completely (= 5)]. Information on family closeness, cohesion, conflict, and cultural involvement were extracted from the Parent Family Environment Scale-Family Conflict Subscale Modified from PhenX (all were represented by binary variables, see S1 Table in S1 File for coding) and the aggregate Sum Scores Culture and Environment Parent instrument (a continuous measure estimated from true/false responses in the previous survey). Additional parent-reported information on child religious affiliation [represented by a binary variable: 1 = any religious preference, 0 = atheist/agnostic or no religion], frequency of religious attendance [an ordinal variable, from never (= 0) to more than once a week (= 4)], and importance of religious beliefs in the child’s daily life [a Likert scale, from not at all (= 1) to very much (= 4)] were extracted from the Longitudinal Parent Demographics Survey. Questions on parent connection to their ethnic group were extracted from the Parent Multi-Group Ethnic Identity-Revised Survey [a Likert scale, from strongly agree (= 1) to strongly disagree (= 5)].

Youth-reported information on parental monitoring was extracted from the Parental-Monitoring Survey [a Likert scale, from never (= 1) to always/almost always (= 5)]. Youth-reported information on caregiver warmth was calculated from the Children’s Report of Parental Behavior Inventory [as the median of all answered responses, each on a Likert scale, from not like him/her (= 1) to a lot like him/her (= 3)]. Information on whether the child lives full-time with the primary caregiver (represented by a binary variable) was extracted from the (Longitudinal) Parent Diagnostic Interview for DSM-5 Background Items Full. The ABCD Family History Assessment Part 1 and the (Longitudinal) Parent Demographics Survey provided information on the number of siblings and number of people living at home, respectively.

#### 2b-2. Parent history of drug/alcohol/mental health problems

The Parent Family History Assessment Part 1 questionnaire provided information on Parent history of drug, alcohol, and mental health problems. Data about both parents was combined into a single ordinal variable representing the number of parents with a history of the problem (0–2).

#### 2b-3. School, neighborhood and community, and peer relationship data

Information on youth attitudes towards school and teachers was obtained from the School Risk and Protective Factors Survey [Likert scale, from definite no (= 1) to definite yes (= 4)]. School setting was extracted from the (Longitudinal) Parent Diagnostic Interview for DSM-5 Background Items Full, and was represented by three indicator variables for in-person school (public, private, vocational, charter, or specialized school), virtual school, and homeschool, respectively. Peer-specific data were obtained from the Longitudinal Parent Diagnostic Interview for DSM-5 Background Items Full (whether the child has a best friend, regular group of friends, problems with bullying, all represented by binary (yes/no) variables), Parent Child Behavior Checklist Raw Scores [Likert scale, from not true (= 0) to very true/often true (= 2)], and the ABCD Cyber Bully (victim of cyberbullying, represented by a binary variable). Although not specific to peers, the ABCD Youth Discrimination Measure also provided data about feeling discriminated against based on weight, sexual orientation, race, ethnicity, and/or being an immigrant (all binary variables). Neighborhood safety and cohesiveness information was extracted from the Parent Neighborhood Safety/Crime Survey Modified from PhenX and the Parent PhenX Community Cohesion Survey [a Likert or reverse Likert scale, from strongly disagree (= 1 or 5) to strongly agree (= 5 or 1)].

#### 2b-4. Youth mental health and temperament

Youth mental health information was extracted from the Parent Diagnostic Interview for DSM-5 Full (KSADS-5). Binary responses to any variables related to anxiety in KSADS-5 were summarized into a single binary variable representing anxiety, and similarly for depression and anhedonia [119]. Fear of social situations and social anxiety disorder diagnosis (past or present) were also represented by binary variables, and similarly for history of trauma. Information related to temperament was extracted from the Parent Diagnostic Interview for DSM-5 Full (KSADS-5) (self esteem and impulsivity, both binary variables), Parent Child Behavior Checklist items (‘would rather be alone’ and ‘self-conscious/easily embarrassed’) were on a Likert scale, from not true (= 0) to very true/often true (= 2), and Parent Child Behavior Checklist Scores Aseba (on internalizing and externalizing behaviors), were continuous measures, with a maximum of 100.

#### 2b-5. Other participant data

Sleep duration information was extracted from the Sleep Disturbance Scale for Children (SDSC) as 1 = 9–11 h; 2 = 8–9 h; 3 = 7–8 h; 4 = 5–7 h; and 5 = less than 5 h. Total screen time (based on information from the Parent Screen Time survey) was calculated as the sum of the number of minutes per weekday multiplied by five and the number of minutes per weekend day multiplied by two. Number of sports was calculated from the (Longitudinal) Parent Sports and Activities Involvement Questionnaire by summing all sports that participants were involved in and number of group activities was calculated by summing the total number of organized group activities that participants were involved in at or outside of school. Physical activity (from the Youth Risk Behavior Survey Exercise Physical Activity) was represented as the number of days the child was physically active for ≥60 minutes per day during the past week [120]. Height and weight data from Youth Anthropometrics Modified from PhenX was used to calculate BMI. Note that not all surveys were available for both baseline and 2-year follow-up (S1 Table in S1 File).

### 2c. Measures of empathetic behaviors and emotions, and CU traits/behaviors

No questions in the ABCD dataset directly measured specific aspects of empathy. Therefore, only questions related to behaviors reflecting empathetic behaviors and emotions, were extracted from parent reports. Three questions were extracted from the Parent Prosocial Behavior Survey (a subset of the Strengths and Difficulties Questionnaire), and were available both at baseline and follow-up: 1) *My child is considerate of other people’s feelings*, 2) *My child is helpful if someone is hurt*, *upset*, *or feeling ill*, and 3) *My child often offers to help others (parents*, *teachers*, *other children)*. One question, more closely related to lack of emotional empathy and CU traits, was taken from the Parent Child Behavior Checklist, and was available at both assessment points: *Doesn’t seem to feel guilt after misbehaving*. Responses to all four above questions were on a Likert scale from not true (= 0) to very true/often true (= 2). At follow-up but not baseline, some additional questions related to empathetic behaviors and CU traits and behaviors were available from the Early Adolescent Temperament Questionnaire. Regarding empathetic behavior, one question was extracted: *Likes taking care of other people*. Reflecting CU traits, two additional questions were available: 1) *When angry at someone*, *says things s/he knows will hurt that person’s feelings*, 2) *Makes fun of how other people look*. Responses were measured on a Likert scale from almost always untrue (= 1) to almost always true (= 5). Finally, two youth-reported questions (not available in parent reports) from the Peer Experiences Questionnaire at follow-up were also analyzed, reflecting CU behaviors as well: 1) *I left another kid out of an activity or conversation that they really wanted to be included in* 2) *I did not invite a kid to a party or other social event even though I knew the kid wanted to go*. These were also measured on a Likert scale from never (= 1) to a few times/week (= 5). Each of these questions were separately examined, for two reasons. First, each question assessed a slightly different aspect of empathetic behaviors/CU traits and behaviors. Second, assessing the consistency of results across variable questions was important to ensure their reliability.

### 2e. Statistical analyses

#### 2e-1. Selection of independent variables

A large set of multidomain parameters was examined. Given the high dimension of the independent variable space, a parsimonious set needed to be identified first as the parameter basis in statistical models. Least absolute shrinkage and selection operator (LASSO) regression [121] is a dimensionality redunction (shrinkage) approach that can be used to identity a parsimonious set of statistical model variables and improves model prediction through regularization. For each empathy-related outcome, 100 repetitions of LASSO were performed with 3-fold cross validation. The final set of independent parameters estimated with this approach corresponded to an optimal model with the lowest Akaike Information Criterion (AIC) value. Separate sets were identified for each outcome (empathetic/CU behavior) at each assessment point, and were used in primary analyses. This process is summarized in S1 Fig of S1 File. Depending on the type of outcome, generalized linear or logistic regression models were developed across analyses, and all data were adjusted for site sampling effects. Sex was included as a biological variable across models. Cohen’s f statistic was used to estimate effect size for each parameter of interest that was significantly associated with an outcome. Across analyses, independent variables included in final analyses were missing data for a relatively small number of participants (<10% of the cohort, and typically <5%). Outcome data were missing for <1% of the cohort. Statistical models only included participants with complete data.

#### 2e-2. Primary analyses

**Fig 1:**
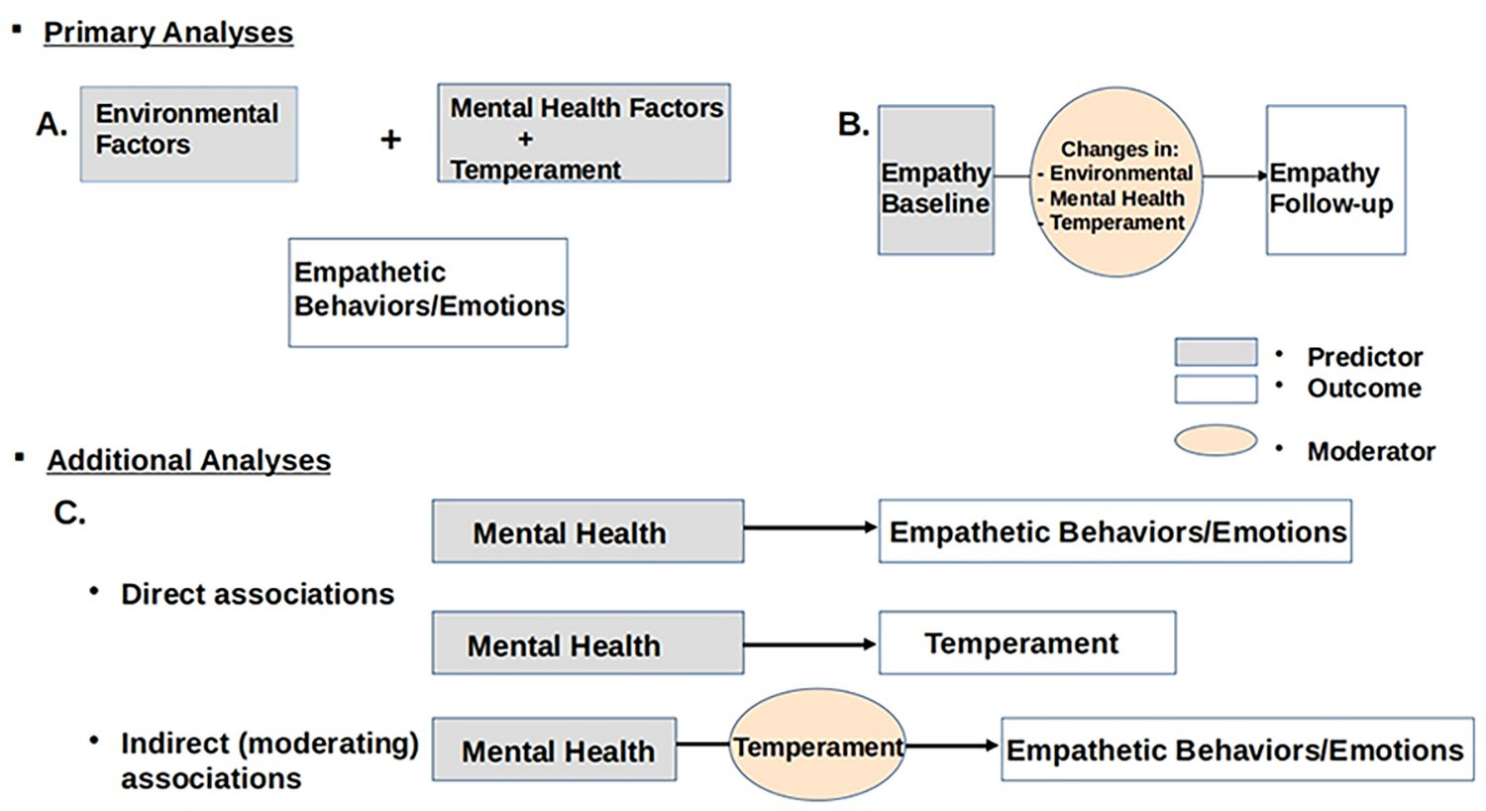
Summary of conducted analyses. Primary analyses (A) were based on the optimal set of environmental, mental health and temperament variables selected through LASSO for each empathetic outcome, separately at baseline and follow-up. Primary analyses (B) investigated the relationship between empathetic behaviors and emotions at the two assessment points and its moderation by changes in environmental, mental health and temperament factors. Additional analyses focused specifically on mental health (particularly anxiety and depression) and correlations with empathetic behaviors/emotions and temperament. The latter’s moderating effect on the relationship between anxiety and depression and empathetic behaviors/emotions was also assessed. Separate analyses were conducted for baseline and follow-up.

The diagrams in Fig 1A and 1B summarize all primary analyses. S2 Table in S1 File lists all independent variables identified by LASSO that were then used in respective analyses.

*Assessment-specific analyses*. To investigate associations between multidomain environmental, mental health, and temperament parameters with empathetic behaviors and emotions, separate statistical models were developed for baseline and 2-year follow-up. Each model used assessment-specific sets of independent parameters identified through LASSO, thus assessing potentially partially distinct domains and parameters correlated with empathy at the two ages. An additional set of models was developed for follow-up analyses, where correlations between independent variables selected at baseline and outcomes at follow-up were examined. The goal of this analysis was to assess potential age-related changes in the significance of parameters and domains linked to empathy at an earlier age.

*Cross-domain interactions*. In addition to models that included only additive parameters from individual domains (main effects), additional models were developed which included interactions between parameters from multiple domains (for example, parental belief in the importance of showing affection and having a best friend).

*Correlations of outcomes between assessments*. For empathetic behaviors/emotions and CU trait/behaviors related outcomes available both at baseline and follow-up, a separate set of analyses examined direct and indirect (moderated) relationships between these outcomes at the two assessments. Models were developed with empathetic behaviors, emotions or CU traits and behaviors as the primary predictors and those at follow-up as the outcomes, including appropriate adjustments for demographic and other variables. First, direct relationships were examined. Then, potential moderating effects of *changes* in environmental, mental health, and temperament factors were considered. Environmental, mental health, and temperament factors included in these models were based on those selected by LASSO at either assessment during primary analysis. Moderations were investigated using a moderated multiple regression (MMR) modeling framework, comparing the additive regression model (which included both the primary predictor and the hypothesized moderator of interest) to the MMR model (which also included the interaction between the predictor and potential moderator) [122–123].

*Adjustment for multiple comparisons and effect sizes*. For each empathetic/CU behavior LASSO identified a somewhat different (though consistent at the domain level) set of independent variables. Therefore, accounting for false discovery could only be achieved by adjusting the level of significance. Thus, instead of α = 0.05, the significance level was set at α = 0.005 (more conservative that a Bonferroni correction across 4 outcomes at baseline–which would correspond to α = 0.0125, or a Bonferroni correction across all 9 outcomes at follow-up–which would correspond to α = 0.0055). In addition, only parameters with at least small effects (Cohen’s f≥ ~0.10, based on rounding from 0.055 to 0.1) are reported.

#### 2e-3. Associations between mental health, environment, and empathetic behaviors/emotions

Statistical models in primary analyses included both environmental and mental health and temperament parameters. Additional statistical models were developed to assess the relationship between anxiety and depression, which are relatively common in adolescence, and empathetic behaviors and emotions, as well as CU traits and behaviors. Following dimensionality reduction, anxiety and depression were not selected by LASSO in any models, possibly due to collinearity with other parameters relatively minor contributions to the models or representation as dichotomous variables. Thus, they were specifically examined in secondary analyses as additional factors of interest (summarized in the diagram in Fig 1C).

## 3. Results

Depending on the measure, ~60 to >90% of the cohort displayed empathetic behaviors and high affective empathy at baseline and follow-up, including statistically more girls than boys (61.33% - 90.00% vs 56.51–86.48%, p<0.01) although the statistical effects of sex were small (Cohen’s f≤ 0.11). In addition, a statistically higher proportion of Hispanic youth was considerate, often offered help, and liked taking care of people compared to Non-Hispanic youth (72.14% - 79.79% vs 69.61% - 75.60%, p≤ 0.03). Differences between racial groups were less consistent. In statistical models, race and ethnicity effects were often statistically nonsignificant (p > 0.05). Table 2 summarizes the distribution of responses on questions related to empathetic behaviors and emotions at the two assessments, and Table 3 the distribution of responses related to CU traits and behaviors.

**Table 2:** 1,2: Distributions of empathetic behaviors and emotions at baseline and the 2-year follow-up assessments. The survey from which relevant parent/youth questions were extracted is also indicated.

**2–1**	**Baseline (N = 11062)**						**Year 2 (N = 9832)**						
	**Parent Prosocial Behavior Survey**												
	**Not True**	**Somewhat True**	**Certainly True**		**Missing**		**Not True**		**Somewhat True**		**Certainly True**		**Missing**
**My child is helpful if someone is hurt, upset, or feeling ill**	185 (1.67%)	1609 (14.55%)	9239 (83.52%)		29 (0.26%)		156 (1.59%)		1564 (15.91%)		8035 (81.72%)		77 (0.78%)
**My child often offers to help others (parents, teachers, other children)**	281 (2.54%)	2515 (22.74%)	8223 (74.33%)		43 (0.39%)		318 (3.24%)		2597 (26.41%)		6840 (69.57%)		77 (0.78%)
**My child is considerate of other people’s feelings**	194 (1.75%)	2409 (21.78%)	8430 (76.21%)		29 (0.26%)		176 (1.79%)		2296 (23.35%)		7283 (74.08%)		77 (0.78%)
**2–2**	**Baseline (N = 11062)**			**Two-year follow-up (N = 9832)**									
	**ABCD Early Adolescent Temperament Questionnaire Parent**												
	Not available			**Almost Always Untrue**		**Usually Untrue**		**Sometimes True/Untrue**		**Usually True**		**Almost Always True**	**Missing**
**Likes taking care of other people**				243 (2.47%)		610 (6.21%)		2831 (28.79%)		3514 (35.74%)		2559 (26.03%)	75 (0.76%)

**Table 3:** 1,2: Distributions of callous/unemotional traits and behaviors at baseline and year-2 follow-up assessments.

**3–1**	**Baseline (N = 11062)**							**Year 2 (N = 9832)**					
	**ABCD Parent Child Behavior Checklist Raw Scores Aseba (CBCL)**												
	**Not True**	**Somewhat/ Sometimes True**	**Very True/ Often True**		**Missing**		**Not True**			**Somewhat/ Sometimes True**		**Very True/ Often True**	**Missing**
**Doesn’t seem to feel guilty after misbehaving**	9738 (88.03%)	1166 (10.54%)	151 (1.37%)		7 (0.06%)		8648 (87.96%)			998 (10.15%)		132 (1.34%)	54 (0.55%)
**3–2**	**Baseline (N = 11062)**			**Year 2 (N = 9832)**									
	**ABCD Early Adolescent Temperament Questionnaire Parent**												
	Not available			**Almost Always Untrue**		**Usually Untrue**			**Sometimes True/Untrue**		**Usually True**	**Almost Always True**	**Missing**
**When angry at someone says things s/he knows will hurt that person’s feelings**				3413 (34.71%)		2452 (24.94%)			2805 (28.53%)		869 (8.84%)	220 (2.24%)	73 (0.74%)
**Makes fun of how other people look**				5184 (52.73%)		2984 (30.35%)			1367 (13.90%)		155 (1.58%)	67 (0.68%)	75 (0.76%)
	**ABCD Peer Experiences Questionnaire**												
	Not available			**Never**		**Once or Twice**			**A Few Times**		**Once a Week**	**Few Times a Week**	**Missing**
**I left another kid out of an activity or conversation that they really wanted to be included in.**				6760 (68.76%)		2475 (25.17%)			501 (5.10%)		33 (0.33%)	42 (0.43%)	21 (0.21%)
**I did not invite a kid to a party/social event though I knew they wanted to go.**				9077 (92.32%)		614 (6.25%)			101 (1.03%)		8 (0.08%)	11 (0.11%)	21 (0.21%)

### 3a. Direct associations between environmental factors and empathetic behaviors and emotions

Associations were examined separately at baseline and follow-up (primary analyses (A) in Fig 1).

#### 3a-1. Common set of independent variables at baseline and follow-up

These analyses aimed to identify significant factors relating to empathetic behaviors and emotions and CU traits and behaviors in pre/early adolescence (baseline) and two years later. Model statistics associated with the results are provided in Tables 4 and 5.

**Table 4:** Associations between environmental, mental health and temperament factors and empathetic behaviors at baseline.

Variable	Regression Coefficient	Cohen’s f	Standard Error (SE)	P-value	Wald Statistic
**Considerate of Others’ Feelings**					
**Important to show affection**	0.05	0.07	0.01	<0.001	47.00
**Frequency of religious service attendance**	-0.03	0.06	<0.01	<0.001	34.85
**Importance of religious beliefs** **in youth’s daily life**	0.03	0.09	0.01	<0.001	33.11
**Has best friend**	0.05	0.07	0.01	<0.001	18.26
**Externalizing behavior score**	-0.02	0.27	<0.01	<0.001	678.29
**Internalizing behavior score**	<0.01	0.07	<0.01	<0.001	50.10
**Would rather be alone**	-0.10	0.08	0.01	<0.001	62.83
**Helpful if Someone is Hurt**					
**Sex**	0.05	0.07	0.01	<0.001	41.67
**Importance of religious beliefs** **in youth’s daily life**	0.047	0.08	0.01	<0.001	57.90
**Religion should be an important part of one’s life**	-0.03	0.07	<0.01	<0.001	44.19
**Parent English Fluency**	0.05	0.07	0.01	<0.001	35.14
**Externalizing behavior score**	-0.01	0.14	<0.01	<0.001	198.55
**Would rather be alone**	-0.07	0.06	0.01	<0.001	38.60
**Often Offers to Help Others**					
**Sex**	0.10	0.10	0.01	<0.001	101.58
**Parents should teach children to be independent**	0.03	0.06	0.01	<0.001	31.94
**Importance of religious beliefs in youth’s daily life**	0.04	0.10	<0.01	<0.001	71.65
**Likes school**	0.03	0.06	0.01	<0.001	40.62
**Externalizing behavior score**	-0.01	0.15	<0.01	<0.001	228.36

**Table 5:** Associations between environmental, mental health and temperament factors and empathetic behaviors at follow-up using independent variables identified at baseline.

Variable	Regression Coefficient	Cohen’s f	Standard Error (SE)	P-value	Wald Statistic
**Considerate of Others’ Feelings**					
**Sex**	0.06	0.07	0.01	<0.001	35.10
**Important to show affection**	0.05	0.07	0.01	<0.001	36.40
**Importance of religious beliefs in youth’s daily life**	0.02	0.07	0.01	0.001	11.40
**Has best friend**	0.06	0.08	0.01	<0.001	20.65
**Externalizing behavior score**	-0.02	0.33	<0.01	<0.001	668.87
**Internalizing behavior score**	<0.01	0.06	<0.01	<0.001	24.14
**Would rather be alone**	-0.08	0.07	0.01	<0.001	34.39
**Helpful if Someone is Hurt, Upset or Ill**					
**Sex**	0.07	0.09	0.01	<0.001	49.39
**Important to show affection**	0.06	0.10	0.01	<0.001	57.51
**Importance of religious beliefs in youth’s daily life**	0.05	0.09	0.01	<0.001	37.61
**Externalizing behavior score**	-0.01	0.16	<0.01	<0.001	160.83
**Fearful of social situations**	-0.09	0.06	0.02	<0.001	23.17
**Often Offers to Help Others**					
**Sex**	0.11	0.11	0.01	<0.001	71.82
**Age**	-<0.01	0.08	<0.01	<0.001	38.08
**Important to show affection**	0.05	0.06	0.01	<0.001	26.63
**Importance of religious beliefs in youth’s daily life**	0.04	0.09	0.01	<0.001	41.33
**Likes school**	0.04	0.07	0.01	<0.001	28.45
**Externalizing behavior score**	-0.01	0.20	<0.01	<0.001	247.95
**Fearful of social situations**	-0.11	0.06	0.02	<0.001	24.21

Overall, girls were more likely to be considerate and offer help than boys (p<0.001, f = 0.07–0.11). The strength of parental belief in the importance of showing love and affection towards one another was positively associated with youth being considerate of others’ feelings at both timepoints, and with being helpful if someone is hurt, upset, or ill and often offering to help others at follow up as well (p<0.001, f = 0.06–0.10). Having a best friend and liking school were consistently positively associated with being considerate and often offering to help, respectively (p<0.001, f = 0.06–0.08). The importance of religious/spiritual beliefs in the youth’s daily life was also consistently positively associated with empathetic behaviors at both assessments (p< 0.001, f = 0.07–0.1). However, the strength of the parental belief that religion should be an important part of one’s life and increased frequency of religious service attendance—both significant at baseline but not follow-up, were negatively associated with being considerate and offering help (p<0.001, f = 0.06–0.07). Additional associations with demographic and other variables are summarized in Tables 4 and 5.

**Fig 2:**
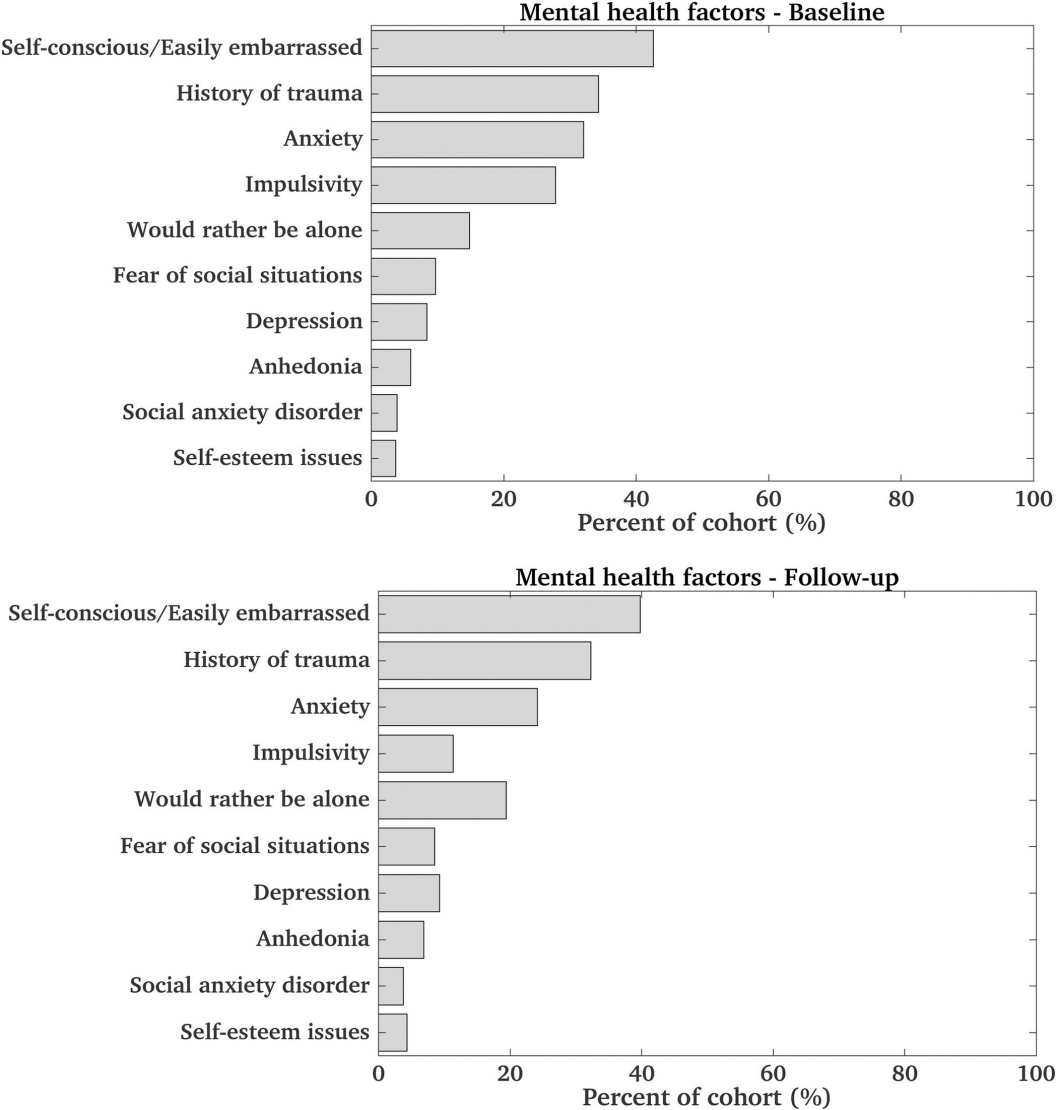
Distribution of mental health factors in the cohort at baseline (top) and follow-up (bottom). In both bar graphs, these factors are sorted based on prevalence at baseline. For factors associated with questions on a scale ‘Not true’, ‘Sometimes true’, ‘Always true’, prevalence for the combined sometimes and always true responses is provided. Note that anxiety and depression values may be an overestimate due to ABCD coding issues. For factors extracted from the parent diagnostic interview, yes to any related questions (for past or present) was assumed as a positive response.

*Youth mental health and temperament*. Distributions of mental health factors and prevalence in the cohort at baseline and follow-up are provided in Fig 2 and S3 Table in S1 File. Higher externalizing behavior scores were consistently negatively correlated with empathetic behaviors and emotions, and their effects were in some cases medium (p<0.001, f = 0.14–0.33). In contrast, higher internalizing behavior scores were positively associated with being considerate (p<0.001, f = 0.06–0.07). Preferring to be alone was negatively associated with being considerate and helpful (the latter only at baseline) and being fearful of social situations was negatively associated with being helpful and offering help at follow-up (p< 0.001, f = 0.06–0.08).

#### 3a-2. Analyses at follow-up using a separate set of independent variables

Additional surveys were available at follow-up. Thus, sets of independent variables were also separately extracted from the larger parameter space at this assessment. The analyses aimed to examine the importance of same aspects of the youth environment as a function of age. Model statistics are summarized in Table 6.

**Table 6:** Associations between environmental, mental health and temperament factors and empathetic behaviors follow-up, using independent variables from the follow-up parameter space.

Variable	Regression Coefficient	Cohen’s f	Standard Error (SE)	P-value	Wald Statistic
**Considerate of Others’ Feelings**					
**Sex**	0.06	0.07	0.01	<0.001	30.29
**Number of sports/activities that youth participates in organized group**	0.02	0.07	<0.01	<0.001	26.82
**Family talks about personal problems**	0.11	0.07	0.02	<0.001	32.28
**Family gets along**	0.07	0.06	0.02	<0.001	19.59
**Parent history of alcohol issues**	0.05	0.06	0.01	<0.001	14.29
**Importance of religious beliefs in youth’s daily life**	0.02	0.09	0.01	<0.001	14.00
**Has best friend**	0.06	0.06	0.01	<0.001	18.18
**Externalizing behavior score**	-0.02	0.33	<0.01	<0.001	668.52
**Internalizing behavior score**	<0.01	0.08	<0.01	<0.001	43.64
**Withdrawn**	-0.12	0.06	0.02	<0.001	24.63
**Helpful if Someone is Hurt, Upset, or Feeling Ill**					
**Sex**	0.06	0.08	0.01	<0.001	32.27
**Family cohesion score**	0.02	0.07	<0.01	<0.001	24.26
**Important to show affection**	0.04	0.07	0.01	<0.001	23.21
**People in this neighborhood do not share the same values** ^*^	0.02	0.06	0.01	<0.001	12.79
**Externalizing behavior score**	-0.01	0.18	<0.01	<0.001	153.29
**Internalizing behavior score**	<0.01	0.06	<0.01	<0.001	18.79
**Often Offers to Help Others**					
**Sex**	0.10	0.10	0.01	<0.001	65.488
**Family income**	-0.02	0.09	<0.01	<0.001	25.13
**Family cohesion score**	0.03	0.08	<0.01	<0.001	41.19
**Importance of religious beliefs in youth’s daily life**	0.04	0.07	0.01	<0.001	19.49
**Externalizing behavior score**	-0.01	0.23	<0.01	<0.001	326.66
**Would rather be alone**	-0.08	0.06	0.02	<0.001	24.95
**Likes taking care of other people**					
**Sex**	0.21	0.11	0.02	<0.001	79.07
**Important to show affection**	0.13	0.08	0.02	<0.001	42.29
**Importance of religious beliefs in youth’s daily life**	0.06	0.06	0.02	<0.001	14.16
**Likes school**	0.06	0.06	0.01	<0.001	19.37
**People in this neighborhood do not share the same values** ^*^	0.04	0.08	0.01	0.003	8.71
**Externalizing behavior score**	-0.02	0.18	<0.01	<0.001	186.49
**Would rather be alone**	-0.21	0.09	<0.03	<0.001	51.07

*The question on neighborhood was asked on a scale where a higher number indicated a higher level of disagreement with the statement.

In addition to factors that were also significantly associated with empathetic behaviors in the previous sets of models (sex, importance of showing affection, having a best friend, importance of religious beliefs, liking school, preferring to be alone, and externalizing and internalizing behaviors), additional family and neighborhood factors were identified. Specifically, having a more cohesive family environment and family who get along with each other and share personal problems was positively associated with being considerate, being helpful, and often offering to help others (p<0.001, f = 0.06–0.08). Furthermore, being in a neighborhood where people shared the same values as the youth’s family was positively associated with being helpful and liking to take care of others (p< 0.01, f = 0.06–0.08). Parent history of alcohol issues (reported in ~14% of parents at both assessments) was also positively associated with being considerate (p< 0.001, f = 0.06). In contrast, being withdrawn was negatively associated with being considerate of other people’s feelings (p< 0.001, f = 0.06). The complete list of associations is provided in Table 6.

### 3b. Direct associations between environmental factors and CU behaviors/traits

Multidomain factors were also associated with CU behaviors, both at baseline and follow-up. Statistical model results are summarized in Table 7.

**Table 7:** Associations between environmental, mental health and temperament factors and CU traits and behaviors at baseline and follow-up.

Variable	Regression Coefficient	Cohen’s f	Standard Error (SE)	P-value	Wald statistic
**BASELINE**					
**Does Not Seem to Feel Guilty After Misbehaving**					
**Externalizing behavior score**	0.02	0.40	<0.01	<0.001	1457.34
**Internalizing behavior score**	-<0.01	0.07	<0.01	<0.001	44.13
**Impulsive**	0.08	0.10	0.01	<0.001	94.78
**FOLLOW-UP USING SET OF INDEPENDENT PARAMETERS IDENTIFIED AT BASELINE**					
**Does Not Seem to Feel Guilty After Misbehaving**					
**Externalizing behavior score**	0.02	0.44	<0.01	<0.001	1296.3
**Internalizing behavior score**	-<0.01	0.07	<0.01	<0.001	26.93
**Anhedonia**	0.14	0.09	0.02	<0.001	61.17
**Impulsive**	0.09	0.11	0.01	<0.001	78.55
**FOLLOW-UP USING SET OF INDEPENDENT PARAMETERS IDENTIFIED AT FOLLOW-UP**					
**Does Not Seem to Feel Guilty After Misbehaving**					
**Parent history of drug issues**	-0.04	0.10	0.01	<0.001	25.87
**Externalizing behavior score**	0.02	0.42	<0.01	<0.001	107115
**Internalizing behavior score**	-0.01	0.08	<0.01	<0.001	37.61
**Anhedonia**	0.09	0.06	0.02	<0.001	21.78
**Impulsive**	0.09	0.07	0.01	<0.001	34.98
**When angry at someone, says things s/he knows will hurt that person’s feelings**					
**Family size**	0.06	0.10	0.01	<0.001	56.86
**Family conflict score**	0.06	0.09	<0.01	<0.001	61.38
**Externalizing behavior score**	0.05	0.44	<0.12	<0.001	1399.07
**I left another kid out of an activity or conversation that they really wanted to be included in**					
**Gets along with teachers**	-0.11	0.10	0.01	<0.001	80.84
**Has regular group of kids to hang out with at school/neighborhood**	0.11	0.07	0.02	<0.001	27.12
**Number of friends**	<0.01	0.06	<0.01	<0.001	26.89
**Being cyberbullied**	0.17	0.07	0.02	<0.001	45.00
**Discriminated against because of race, ethnicity and/or skin color**	0.19	0.07	0.03	<0.001	34.10
**I did not invite a kid to a party or other social event even though I knew the kid wanted to go**					
**Gets along with teachers**	-0.03	0.06	0.01	<0.001	34.59
**Being cyber-bullied**	0.08	0.07	0.01	<0.001	42.51

Higher externalizing behavior scores and being impulsive were consistently associated with higher likelihood of not feeling guilty when misbehaving at both assessment points, while the opposite association was estimated for internalizing behaviors (p<0.001, f = 0.40–0.44 for externalizing, f = 0.07–0.11 for impulsive, f = 0.07–0.08 for internalizing). Having anhedonia was also positively associated with lack of guilt but only at follow-up (p<0.001, f = 0.06–0.09). In contrast, parent history of drug problems (reported in >20% of parents at both assessments) was correlated with being more likely to feel guilty after misbehaving at follow-up (p<0.01, f = 0.10). Furthemore, a larger family and higher family conflict were associated with being more likely to say things to hurt others’ feelings (p<0.001, f = 0.09–0.10). Being cyberbullied or discriminated against because of race, ethnicity, or skin color was associated with a higher likelihood of CU behaviors, such as leaving a kid out from an activity or not inviting a kid to a social event they really wanted to be part of (p<0.001, f = 0.07). Having more friends or a regular group of kids to hang out with was also associated with a higher likelihood of leaving kids out of an activity they wanted to be part of (p<0.001, f = 0.06–0.07). In contrast, getting along with teachers was associated with a lower likelihood of these callous behaviors (p<0.001, f = 0.06–0.10).

### 3c. Multidomain interactions

Interactions between externalizing behavior scores and impulsivity, and similarly between externalizing and internalizing scores, were positively associated with not feeling guilty when misbehaving at both baseline and follow-up (p<0.001, f = 0.10–0.24). In addition, at follow-up, interactions between anhedonia and externalizing scores and anhedonia and impulsivity were positively associated with not feeling guilty (p<0.001, f = 0.10–0.15).

### 3d. Correlations of empathetic/CU behaviors between assessments

Temporal relationships between empathetic/CU behaviors were also examined (primary analyses (B) in the diagrams in Fig 1). Models were first adjusted only for demographic and other participant data, but no environmental, mental health, or temperament parameters. Significant positive temporal correlations were estimated for all empathetic outcomes (p<0.001, f = 0.37–0.51). These are summarized in Table 8A. Then, changes in mental health, temperament, and environmental factors were also incorporated in the models. Significant changes impacting the correlation between behaviors at the two assessments are summarized in Table 8B. Their moderating effects were then assessed using the MMR framework. Sex (specifically, being a girl) was a significant contributor to temporal correlations between empathetic behaviors (p<0.001). Increased externalizing behavior scores at follow-up (relative to baseline) negatively moderated the temporal relationship of being considerate of other people’s feelings (p<0.005, moderation effect size = 0.12), and positively moderated that of not feeling guilt after misbehaving (p<0.005, moderation effect size = 0.25). Increased impulsivity also positively moderated the latter (p<0.005, moderation effect size = 0.04).

**Table 8:** Direct associations between empathetic/CU behaviors at baseline and follow-up (8a), with only demographic adjustments. Direct associations between behaviors with additional adjustments for changes in environmental, mental health and/or temperament factors (8b).

**(8a)**					
**Empathetic/CU behaviors/emotions at each assessment**	**Standardized Regression Coefficient**	**Cohen’s f**	**Standard Error (SE)**	**P-Value**	**Wald Statistic**
**Considerate of Others’ Feelings**	0.45	0.51	0.01	<0.001	2424.07
**Helpful if Someone is Hurt**	0.40	0.43	0.01	<0.001	1737.76
**Often Offers to Help**	0.40	0.44	0.01	<0.001	1800.18
**Doesn’t Seem to Feel Guilty after Misbehaving**	0.35	0.37	0.01	<0.001	1176.37
**(8b)**					
**Independent Variables**	**Regression Coefficient**	**Cohen’s f**	**Standard Error (SE)**	**P-Value**	**Wald Statistic**
**Considerate of Others’ Feelings (follow-up)**					
**Considerate of others’ feeling (baseline)**	0.48	0.52	0.01	<0.001	1474.46
**Sex**	0.06	0.07	0.01	<0.001	27.22
**Change in externalizing behavior score**	-0.01	0.11	<0.01	<0.001	67.92
**Helpful if Someone is Hurt (follow-up)**					
**Helpful if someone is hurt (baseline)**	0.39	0.42	0.01	<0.001	924.99
**Sex**	0.05	0.06	0.01	<0.001	20.98
**Change in externalizing behavior score**	-<0.01	0.06	<0.01	<0.001	22.12
**Often Offers to Help Others (follow-up)**					
**Often Offers Help (baseline)**	0.45	0.47	0.01	<0.001	1371.54
**Sex**	0.07	0.08	0.01	<0.001	36.48
**Age**	-<0.01	0.07	<0.01	<0.001	31.21
**Change in importance of religious beliefs in the youth’s daily life**	0.03	0.08	0.01	<0.001	15.72
**Change in externalizing behavior score**	-0.01	0.07	<0.01	<0.001	33.96
**Doesn’t Seem to Feel Guilty After Misbehaving**					
**No Guilt After Misbehaving (baseline)**	0.43	0.46	0.02	<0.001	1313.48
**Change in externalizing behavior score**	0.01	0.24	<0.01	<0.001	375.15
**Change in anhedonia status**	0.07	0.06	0.02	<0.001	22.65
**Change in impulsivity** ^*^	0.05	0.06	0.01	<0.001	24.17

*Impulsivity was assessed by the CBCL.

### 3e. Secondary analyses: Depression, anxiety and empathetic/CU behaviors

Although several mental health and temperament factors were significantly associated with empathetic/CU behaviors in primary analyses, depression and anxiety (both relatively common in adolescence) were not selected by LASSO for any models, potentially as the result of their correlation with other variables. Given their prevalence in adolescence, the associations between anxiety (affecting over 30% of youth in this cohort at baseline and almost 25% at follow-up) and depression (affecting almost 10% of youth at both assessments) and empathetic behaviors and emotions were thus separately examined (analyses C in the diagrams in Fig 1).

At both assessments, having anxiety or depression was correlated with being less considerate of others’ feelings and less likely to be helpful/offer help (p < 0.001, f = 0.06–0.14), independently of environmental factors. At baseline, both were associated with being less likely to feel guilty after misbehaving (p < 0.001, f = 0.09–0.12). At follow-up, anxiety and depression were positively associated with saying things to hurt others’ feelings when angry (and the latter with making fun of how people look as well) and negatively associated with liking to take care of others (p<0.001, f = 0.06–0.20). These results are summarized in S4 Table of S1 File.

Both anxiety and depression were also positively associated with externalizing and internalizing scores at both assessment points (p < 0.001, f = 0.17–0.41). Internalizing and externalizing scores were also examined as potential moderators of the relationship between anxiety/depression and empathetic/CU behaviors. At baseline and follow-up, internalizing behavior scores negatively moderated the relationship between depression and being considerate (moderation effect size ≤ 0.13). At follow-up, internalizing behaviors also significantly positively moderated the relationship between depression and not seeming to feel guilty after misbehaving (moderation effect size = 0.16). No other statistical moderations were estimated.

## 4. Discussion

Empathy is a critical aspect of our social world. Although humans may have some capacity for empathetic concern even at birth [124–126], empathy develops over time, particularly in the first two decades of life, and is affected by myriads of genetic, environmental, and experiential factors [14, 26, 42–44, 127, 128]. The effects of the youth environment on the development of empathy, particularly during biologically and socially complex periods such as adolescence, are incompletely understood. This is in part due to the heterogeneity of development but also the significant variability of empathy in youth [45] and the evolving neural circuits that support it [129, 130].

In this first-of-its-kind study in size and scope, we have leveraged early longitudinal data from the ABCD study on parents, family, school, peers, community/neighborhood, culture, and religiosity, as well as mental health and temperament, with the overarching goal to identify significant environmental and mental health predictors of the development of empathetic behaviors in pre and early adolescence. In the context of the ecological systems theory, analyzed factors and their interactions span three system levels (micro to macro) in the youth social environment. We hypothesized individual and combinatorial relationships between these factors and the development of empathetic behaviors and emotions. To test this hypothesis, we examined empathetic behaviors and meotions (and for comparison CU traits and behaviors) and their temporal changes within a historically large cohort of n = 11062 pre/early adolescents at baseline and n = 9832 at the two-year follow-up. At both assessments, parents reported that at least 70% of youth displayed empathetic behaviors, such as being considerate of other people’s feelings, liking to take care of people, and being helpful to others, including when someone is hurt. In addition, ~50–60% never said hurtful things when angry or made fun of others’ looks, almost 90% felt guilty when misbehaving and at least ~70% would never exclude other kids from activities and events they wanted to be part of. Overall, girls were more likely to display empathetic behaviors than boys.

Associations between a common set of environmental and mental health parameters (identified as significant correlates of empathetic/CU behaviors at baseline) and each outcome of interest at both assessments were first examined in order to determine whether they were consistently significant as a function of development (across a period of two years, from baseline to follow-up). Factors in the youth ecological microsystem were consistently correlated with empathetic behaviors. In particular, parental beliefs on the importance of family members showing affection towards each other was positively correlated with empathetic behaviors at both assessments. When a second set of environmental and mental health factors were separately estimated from the available parameter space at follow-up, additional family-related factors were correlated with these behaviors. Specifically, family cohesion and positive dynamics, with family members getting along and sharing personal problems, was positively linked to empathetic behaviors and emotions. These findings align well with those of prior studies, which have shown that children who grow up with high attachment security and loving and supportive parents in a positive family environment have higher empathy and prosocial behaviors than those who experience negative/rejecting and neglectful parenting and negative family dynamics [100, 131–139]. In addition, meta-analyses have shown that understanding others’ emotions is specifically correlated with the shared family environment [139]. In contrast, high family conflict was associated with CU behaviors, such as saying hurtful things when angry. Family/parent hostility, anger, and violence may have profound detrimental effects on children’s social development [140–143] and have been shown to adversely affect youth mental health and temperament [144] and impair emotion processing and regulation, which may, in turn, disrupt the development of empathy. Children learn to regulate their emotions (including empathetic ones) in part through observation, and this process may be adversely impacted by experiencing family conflict [145].

Caregiver history of substance use may also have direct and indirect effects on the development of empathy in youth [146]. In this cohort, more than 20% of caregivers reported a history of substance use issues, which were correlated with the behaviors of interest but only at follow-up, i.e., in older youth. Specifically, parent history of alcohol issues was positively correlated with being considerate of other people’s feelings, while parent history of drugs was associated with being more likely to feel guilty after misbehaving. Parental substance use may have a profound effect on youth emotional, behavioral, and mental health, and temperament [147–149]. However, children of parents who abuse alcohol and drugs may also engage in role reversal, i.e., parentification, which, despite its adverse impact on youth mental health, may also be associated with increased empathy [150].

Relationships with peers, who are also part of the youth ecological microsystem, were also identified as significant contributors to empathetic behaviors. Having a best friend was consistently positively associated with being more considerate across assessments, while a large number of friends and/or having a regular group of friends to hang out with were correlated with more frequent peer exclusion at follow-up. As the youth social world expands in adolescence, high-quality peer relationships may help shape cognitive and affective empathy and prosocial motivation [57, 151–153]. However, having large groups of friends could dilute the positive effects of high-quality friendships, and the intensity of connectedness within a social group. Being part of a homogeneous/close group of friends may also lead to peer exclusion [151, 154–159]. Effect sizes of peer relationship factors were not statistically different than those of family factors at follow-up, suggesting that the impact of social reorientation may not be measurable from pre to early adolescence or may not be correlated with the behaviors examined here. In terms of negative peer experiences, being cyberbullied (>8% of the cohort at follow-up) and feeling discriminated against based on weight, race, ethnicity, or skin color (~5% of the cohort at follow-up) were each correlated with more frequent peer exclusion. A number of studies have shown that victims of bullying are at higher risk of developing mental health issues, including anxiety, depression, and internalizing problems [152]. Here, in agreement with prior studies [153], higher internalizing behaviors were positively associated with empathetic behaviors, suggesting an indirect effect of bullying and/or some forms of discrimination on empathy. Finally, we also examined school/teacher-related factors, another aspect of the youth ecological microsystem. Liking school was consistently associated with often offering to help others at both assessments (and liking to take care of others at follow-up), while getting along with teachers was associated with being less likely to exclude other youth from events/activities, in agreement with prior studies [42, 62–64]. Together, these findings suggest that across domains of the youth microsystem, positive factors associated with nurturing caregivers, a cohesive family environment, close peer relationships, positive teacher-student relationships, and positive attitudes towards the school environment are significant contributors to the development of empathetic behaviors and emotions.

At the ecological macrosystem, the importance of religious/spiritual beliefs in the youth’s life was also consistently positively associated with empathetic behaviors across assessments. In contrast, frequency of religious service attendance and the parental belief that religion should be an important part of one’s life were negatively correlated with some of these behaviors, though infrequently. Our findings are in agreement with prior work, including studies on adolescents, reporting positive associations between empathy and importance of religion or religious commitment but not religious involvement [79, 80]. Associations between other aspects of the macrosystem, such as culture and community, and youth empathy were more difficult to assess, in part because some related surveys were only available at follow-up. Nonetheless, living in a neighborhood where others shared their parents’ values was positively correlated with being helpful and taking care of people. This finding is aligned with prior work showing that neighborhood cohesion is correlated with the development of prosocial behaviors and emotional regulation in youth [160, 161].

Mental health and temperament factors and their interactions were also significantly correlated with the outcomes of interest. Higher externalizing behaviors were negatively associated with empathetic behaviors and positively associated with CU traits and behaviors across assessments, while the opposite relationships were estimated for internalizing behaviors. Preferring to be alone, being withdrawn, and being fearful of social situations were often negatively associated with empathetic behaviors. Secondary analyses also revealed that anxiety and depression were negatively correlated with empathetic behaviors and positively associated with CU traits and behaviors. Being impulsive and having anhedonia were, however, positively associated with not feeling guilty when misbehaving. In addition, at both assessments, interactions between externalizing and internalizing behaviors, and externalizing behaviors and impulsivity were positively associated with not feeling guilt. At follow-up, additional interactions between anhedonia and externalizing behaviors, and internalizing behaviors and impulsivity were also positively related to lack of guilt. The effects of externalizing behaviors, individually and in some combinations with other mental health factors (interactions), were notably larger than all others. Our findings are in agreement with extensive prior reports of negative correlations between externalizing behaviors, social withdrawal, anxiety, depression, and prosocial behaviors, but positive associations between internalizing behaviors and empathy [39, 87, 88, 162, 163].

Finally, to elucidate developmental changes in empathetic/CU behaviors and the effects of the temporally varying youth environment, we examined temporal correlations between these behaviors and the moderating effects of changes in environmental and mental health and temperament factors. Although there were some changes in environmental factors, such as in the reported strength of certain parental beliefs and importance of religion in the youth’s life, these did not moderate the correlation between any of the behaviors of interest across the two assessments. However, increased externalizing behavior scores at follow-up (relative to baseline) negatively moderated the temporal relationship of being considerate of other people’s feelings and positively moderated that of not feeling guilt after misbehaving. The latter was also moderated by increased impulsivity.

Despite its many strengths, this study also had some limitations. First, it is a retrospective investigation of data collected for purposes not directly related to empathy. Although relevant information needed to be extracted from multiple instruments, in the aggregate it captured behaviors and emotions related to empathy and CU traits and behaviors. In addition, genetic factors could not be measured. Furthermore, at the time of the study, only early longitudinal data were available. Thus, only limited temporal changes in the youth environment could be assessed, which prevented the assessment of social reorientation, likely a longer process. However, the ABCD study will collect relevant data at multiple assessment points throughout adolescence. Thus, future investigations may characterize the developmental trajectories of empathetic behaviors across longer periods of time. In addition, an extensive correlation analysis between different youth mental health, temperament, and environmental factors was not conducted. Instead, statistical analyses identified the most significant contributors to youth empathetic behaviors, and additional analyses focused on factors that either had large effects on these relationships (e.g., externalizing behaviors) or are common in adolescence or prevalent in the cohort (e.g. anxiety). Furthermore, bidirectional relationships between mental health and empathetic behaviors were not assessed (specifically the effects of the latter on the former), as they were outside the scope of this study. Finally, given limited longitudinal data, a true causal analysis was not possible. Thus, we only examined correlations between empathetic outcomes at the two assessments and the effects of environmental and mental health changes on them.

Based on a historically large adolescent sample with early longitudinal data and an extensive investigation of combined associations between environment and mental health factors and youth empathetic behaviors, this study makes a significant scientific contribution toward our incomplete understanding of how empathy in adolescence is profoundly shaped by these factors. It highlights the importance of showing affection toward the youth, a cohesive and positive family environment, positive peer relationships, a positive attitude towards school and good relationships with teachers, and a cohesive neighborhood that shares the family’s values, but also the importance of religiosity on youth empathy. However, it also highlights that the development of empathy is vulnerable to negative family dynamics, a large number of (presumably not close) friends, bullying victimization, discrimination, and mental health issues, including anhedonia, anxiety, depression, externalizing behaviors, and their interactions. Temporal changes in these mental health factors may also moderate early adolescent trajectories of empathetic behaviors. Overall, our findings suggest that the development of empathetic behaviors and emotions is a complex process that may be critically depend on environmental factors across ecological systems, and is vulnerable to multi-domain risk factors and mental health issues.

## Supporting information

S1 File An additional flow diagram is provided outlining the analysis steps. Four supplemental tables provide information on instruments and variables analyzed in the study and summarize results from secondary analyses.
(DOC)
