# Supplementary material for: Effects of multidomain environmental and mental health factors on the development of empathetic behaviors and emotions in adolescence
Source: PLoS One. 2023 Nov 22;18(11):e0293473. doi: 10.1371/journal.pone.0293473 (PMC10664943; doi:10.1371/journal.pone.0293473)
Supplement: S1 File — Four supplemental tables provide information on instruments and variables analyzed in the study and summarize results from secondary analyses. (DOC) [file pone.0293473.s001.doc]

**SUPPLEMENTAL MATERIALS**

**S-1: Supplemental Figure**


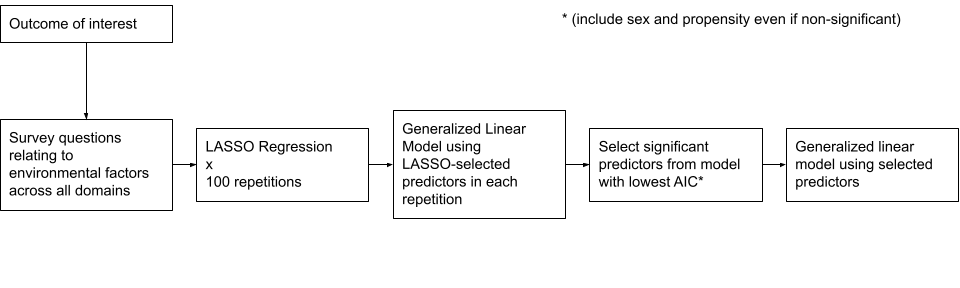


**Figure S1:** *Flow diagram describing the process by which a parsimonious and optimal set of environmental, mental health, temperament and other independent variables are selected for each outcome of interest, using LASSO.*

**S-2. Supplemental Tables**

**Table S1:** *All independent variables given to LASSO for variable selection. Questions are from parent reports unless marked with * for youth report, and only refer to parent’s self statements when noted as parent self-report (P:). Availability at baseline (B) and two-year follow-up (2) is also indicated. Coding for inclusion in models and any necessary changes are indicated in the right-hand column. The surveys from which individual variables was extracted are listed in the second column.*

| **Question** | **Survey** | **Availability** | **Coding** |
| --- | --- | --- | --- |
| **Demographics/Anthropometrics/Physical/Behavioral Data** | | | |
| Sex at birth | (all) | B / 2 | Male, Female, Other (0 responses for other) |
| Interview age | (all) | B / 2 | Continuous in months |
| Race | ABCD Parent Demographics Survey (PDS) | B | Dichotomized: White = 1, Nonwhite =0. If white and another race are selected, count as nonwhite. Assume same values at year 2 |
| Hispanic/Latino | ABCD PDS | B | 1 = Hispanic, 0 = Non-Hispanic. Assume same values at year 2 |
| Total combined family income for the past 12 months | ABCD (Longitudinal) PDS | B / 2 | 1= Less than $5,000; 2=$5,000 through $11,999; 3=$12,000 through $15,999; 4=$16,000 through $24,999; 5=$25,000 through $34,999; 6=$35,000 through $49,999; 7=$50,000 through $74,999; 8= $75,000 through $99,999; 9=$100,000 through $199,999; 10=$200,000 and greater. 999 = Don't know/NaN; 777 = Refuse to answer/NaN |
| Level of education of primary caregiver | ABCD (Longitudinal) PDS | B / 2 | Responses adjusted to be ordinal: High school diploma and GED combined, Associate degrees combined, and higher degrees (PhD, MD) combined |
| Marital status of primary caregiver | ABCD (Longitudinal) PDS | B / 2 | Dichotomized: 1=Married/living with partner, 0 = any other response |
| Number of brothers/sisters | ABCD Family History Assessment Part 1 | B | Sum number of brothers and number of sisters |
| Number of people living at address | ABCD (Longitudinal) PDS | B / 2 | continuous |
| Child lives with primary caregiver full time | ABCD (Longitudinal) Parent Diagnostic Interview for DSM-5 Background Items Full (KSADS-Back) | B / 2 | Not Applicable coded as NaN |
| Years at current address | Residential History Derived Scores | B | continuous |
| UN adjusted population density | Residential History Derived Scores | B | continuous |
| Site ID | ABCD Longitudinal Tracking | B / 2 | In combination with census tract information, two sets of indicator variables (region, division) show whether a participant site is (1) or is not (0) located in each census region and division:  Region:  NE, MidW, S, W  Division:  New England, Mid Atlantic, EN Central, WN Central, S Atlantic, ES Central, WS Central, Mtn, Pacific |
| Standing Height Average (inches) and Weight record 1 (lbs) | ABCD Youth Anthropometrics Modified From PhenX (ANT) | B / 2 | weight/(height^2)*703 |
| Number of hours of sleep | ABCD Parent Sleep Disturbance Scale for Children (SDS) | B / 2 | 1 = 9-11 hours; 2 = 8-9 hours; 3 = 7-8 hours; 4 = 5-7 hours; 5 = Less than 5 hours |
| Time spent on a computer, cellphone, tablet, or other electronic device | ABCD Parent Screen Time Survey (STQ) | B / 2 | 5*((hrs/weekday)*60+(mins/weekday)) + 2*(hrs/weekend day)*60+(mins/weekend day)) |
| Number of days in the past week physically active for at least 60 minutes | ABCD Youth Youth Risk Behavior Survey Exercise Physical Activity (YRB) | B / 2 | 1:7 |
| Number of given sports/activities that youth participated in | ABCD Parent Sports and Activities Involvement Questionnaire (SAIQ) | B / 2 | 1:28 |
| Number of given sports/activities that youth participated in as part of an organized group | ABCD (Longitudinal) Parent Sports and Activities Involvement Questionnaire (SAIQ) | B / 2 | 1:28 |
| **Family Dynamics** | | | |
| We fight a lot in our family. | ABCD Parent Family Environment Scale-Family Conflict Subscale Modified from PhenX (FES) | B / 2 | 1=True, 0=False |
| Family members rarely become openly angry | PhenX (FES) | B / 2 | 0=True, 1=False |
| Family members sometimes get so angry they throw things | PhenX (FES) | B / 2 | 1=True, 0=False |
| Family members hardly ever lose their tempers | PhenX (FES) | B / 2 | 0=True, 1=False |
| Family members often criticize each other. | PhenX (FES) | B / 2 | 1=True, 0=False |
| Family members sometimes hit each other. | PhenX (FES) | B / 2 | 1=True, 0=False |
| If there is a disagreement in our family, we try hard to smooth things over and keep the peace. | PhenX (FES) | B / 2 | 0=True, 1=False |
| Friends often come over for dinner or to visit | PhenX (FES) | 2 | 1=True, 0=False |
| There is a feeling of togetherness in our family | PhenX (FES) | 2 | 1=True, 0=False |
| We tell each other about our personal problems | PhenX (FES) | 2 | 1=True, 0=False |
| We are not that interested in cultural activities. | PhenX (FES) | 2 | 0=True, 1=False |
| Family members really back each other up | PhenX (FES) | 2 | 1=True, 0=False |
| Family members really help and support one another | PhenX (FES) | 2 | 1=True, 0=False |
| There is very little group spirit in our family | PhenX (FES) | 2 | 0=True, 1=False |
| We really get along well with each other. | PhenX (FES) | 2 | 1=True, 0=False |
| Family members often keep their feelings to themselves | PhenX (FES) | 2 | 0=True, 1=False |
| There is plenty of time and attention for everyone in our family | PhenX (FES) | 2 | 1=True, 0=False |
| Cohesion subscale From the FamIly Environment Scale Sum of Parent Report Prorated Score (Based on minimum of 5 Items answered) | ABCD Sum Scores Culture & Environment Parent | 2 | continuous |
| Conflict subscale from the Family Environment Scale Sum of Parent Report Prorated Score (Based on minimum of five items answered) | ABCD Sum Scores Culture & Environment Parent | B / 2 | continuous |
| How often parents/guardians know where youth is * | ABCD Parental Monitoring Survey (PMS) | B / 2 | 1 = Never; 2 = Almost Never; 3 = Sometimes; 4 = Often; 5 = Always or Almost Always |
| How often parents know who youth is with when not at school or home * | ABCD PMS | B / 2 | 1:5 |
| If at home when parents are not, how often youth knows how to get in touch * | ABCD PMS | B / 2 | 1:5 |
| How often youth talks to parents about plans for the coming day (what will happen at school, what they are going to do with friends) * | ABCD PMS | B / 2 | 1:5 |
| Frequency of eating dinner with parents per week * | ABCD PMS | B / 2 | 1:5 |
| (primary caregiver) Makes me feel better after talking over my worries, smiles at me very often, is able to make me feel better when I am upset, believes in showing his/her love for me, is easy to talk to * | ABCD Children's Report of Parental Behavioral Inventory | B / 1 | Median of all responses to these questions (1 = Not like him/her; 2 = Somewhat like him/her; 3 = A lot like him/her) |
| **Parent History of Mental Health and Substance Abuse Issues** | | | |
| Parents alcohol problem | ABCD Parent Family History Summary Scores (PFHS) * | B | Recoded ordinally: 0= none; 1 = either parent; 2 = both parents |
| Parents drug use problem | ABCD PFHS | B | 0:2 |
| Parents depression problem | ABCD PFHS | B | 0:2 |
| Parents trouble holds job/fights/police problem | ABCD PFHS | B | 0:2 |
| Parents nerves/nervous breakdown problem | ABCD PFHS | B | 0:2 |
| Parents hospitalized due to emotional/mental problem | ABCD PFHS | B | 0:2 |
| Parents attempted or committed suicide | ABCD PFHS | B | 0:2 |
| - baseline responses from this survey were also used at year 2 as they provide a minimum history | | | |
| **Parent Family Beliefs / Values** | | | |
| P: Parents should teach their children that the family always comes first. | ABCD Parent Mexican American Cultural Values Scale Modified (MACV) | B / 2 | 1 = Not at All; 2 = A Little; 3 = Somewhat; 4 = Very Much; 5 = Completely |
| P: Children should be taught that it is their duty to care for their parents when their parents get old. | ABCD MACV | B / 2 | 1:5 |
| P: Children should always do things to make their parents happy. | ABCD MACV | B / 2 | 1:5 |
| P: People should learn how to take care of themselves and not depend on others. | ABCD MACV | B / 2 | 1:5 |
| P: Family provides a sense of security because they will always be there for you. | ABCD MACV | B / 2 | 1:5 |
| P: If a relative is having a hard time financially, one should help them out if possible. | ABCD MACV | B / 2 | 1:5 |
| P: When it comes to important decisions, the family should ask for advice from close relatives | ABCD MACV | B / 2 | 1:5 |
| P: The most important thing parents can teach their children is to be independent from others. | ABCD MACV | B / 2 | 1:5 |
| P: It is always important to be united as a family. | ABCD MACV | B / 2 | 1:5 |
| P: A person should share their home with relatives if they need a place to stay. | ABCD MACV | B / 2 | 1:5 |
| P: As children get older their parents should allow them to make their own decisions. | ABCD MACV | B / 2 | 1:5 |
| P: It is important to have close relationships with aunts/uncles, grandparents, and cousins. | ABCD MACV | B / 2 | 1:5 |
| P: Older kids should take care of and be role models for their younger brothers and sisters. | ABCD MACV | B / 2 | 1:5 |
| P: Children should be taught to always be good because they represent the family. | ABCD MACV | B / 2 | 1:5 |
| P: When there are problems in life, a person can only count on him or herself. | ABCD MACV | B / 2 | 1:5 |
| P: Holidays and celebrations are important because the whole family comes together. | ABCD MACV | B / 2 | 1:5 |
| P: Parents should be willing to make great sacrifices to make sure their children have a better life. | ABCD MACV | B / 2 | 1:5 |
| P: A person should always think about their family when making important decisions. | ABCD MACV | B / 2 | 1:5 |
| P: Parents should encourage children to solve their own problems. | ABCD MACV | B / 2 | 1:5 |
| P: It is important for family members to show their love and affection to one another. | ABCD MACV | B / 2 | 1:5 |
| P: It is important to work hard and do one's best because this work reflects on the family. | ABCD MACV | B / 2 | 1:5 |
| **Religiosity** | | | |
| Religious preference | ABCD (Longitudinal) PDS | B / 2 | 1=religious preference, 0=atheist, agnostic, or no religion |
| Frequency of religious attendance | ABCD (Longitudinal) PDS | B / 2 | 0 = Never; 1 = Less than once a month; 2 = One to three times a month; 3 = About once a week; 4 = More than once a week; 999 = Don't know/NaN; 777 = Declined/NaN |
| Importance of religious and spiritual beliefs in day-to-day | ABCD (Longitudinal) PDS | B / 2 | 1 = Not at all; 2 = Not very; 3 = Somewhat; 4 = Very; 999 = Don't know/NaN; 777 = Declined/NaN |
| P: One's belief in God gives inner strength and meaning to life | ABCD MACV | B / 2 | 1:5 |
| P: God is first; family is second | ABCD MACV | B / 2 | 1:5 |
| P: Parents should teach their children how to pray. | ABCD MACV | B / 2 | 1:5 |
| P: If everything is taken away, one still has their faith in God. | ABCD MACV | B / 2 | 1:5 |
| P: It is important to thank God every day for all one has | ABCD MACV | B / 2 | 1:5 |
| P: It is important to follow the Word of God. | ABCD MACV | B / 2 | 1:5 |
| P: Religion should be an important part of one's life. | ABCD MACV | B / 2 | 1:5 |
| **Culture** | | | |
| P: strong sense of belonging to my own ethnic group | ABCD Parent Multi-Group Ethnic Identity-Revised Survey (MEIM) | B / 2 | 1 = Strongly Agree; 2 = Agree; 3 = Neutral; 4 = Disagree; 5 = Strongly Disagree |
| P: strong attachment towards my own ethnic group. | ABCD Parent Multi-Group Ethnic Identity-Revised Survey (MEIM) | B / 2 | 1:5 |
| P: spoken English ability | ABCD Parent Acculturation Survey Modified from PhenX (ACC) | B / 2 | 1-Poor; 2-Fair; 3-Good; 4-Excellent; 999-Don't know/NaN; 777-Refused/NaN |
| Spoken English ability* | ABCD Youth Acculturation Survey Modified from PhenX (ACC) | B / 2 | 1-Poor; 2-Fair; 3-Good; 4-Excellent; 999-Don't know/NaN; 777-Refused/NaN |
| **School/Teachers** | | | |
| Students have lots of chances to help decide things like class activities and rules * | ABCD School Risk and Protective Factors Survey (SRFP) | B / 2 | 1 = NO!; 2 = no; 3 = yes; 4 = YES! |
| I get along with my teachers * | ABCD SRFP | B / 2 | 1:4 |
| My teacher(s) notices when I am doing a good job and lets me know about it * | ABCD SRFP | B / 2 | 1:4 |
| Lots of chances for students to get involved in sports, clubs, or other school activities outside of class * | ABCD SRFP | B / 2 | 1:4 |
| I feel safe at my school * | ABCD SRFP | B / 2 | 1:4 |
| Lots of chances to be part of class discussions or activities * | ABCD SRFP | B / 2 | 1:4 |
| In general, I like school a lot * | ABCD SRFP | B / 2 | 1:4 |
| Usually, school bores me * | ABCD SRFP | B / 2 | 1:4 |
| Current school enrollment | ABCD (Longitudinal) KSADS-Back | B / 2 | Converted to 3 binary variables: In person: 1= regular public or private, vocational, charter, specialized, 0 = Any other option; Home schooled: 1=home schooled, 0=Any other option; Virtual: 1 = virtual, 0 = any other option |
| **Peers** | | | |
| Has a regular group of kids they hangs out with at school or in your neighborhood | ABCD (Longitudinal) KSADS-Back | B / 2 | 1 = Yes; 2 = No; 3 = Not sure/NaN; 777 = Decline to answer/NaN |
| Has a best friend | ABCD (Longitudinal) KSADS-Back | B / 2 | 1 = Yes; 2 = No; 3 = Not sure/NaN; 777 = Decline to answer/NaN |
| Number of friends (boys, girls, other) * | ABCD Other Resilience | B / 2 | Sum number of friends of any gender |
| Number of close friends (boys, girls, other) * | ABCD Other Resilience | B / 2 | Sum number of close friends of any gender |
| **Community** | | | |
| This is a close-knit neighborhood. | ABCD Parent PhenX Community Cohesion (CoCo) | 2 | 5= Strongly Agree;4= Agree; 3= Neither Agree Nor Disagree; 2= Disagree; 1 = Strongly Disagree; 999 = Don't Know/NaN; 777= Refused/NaN |
| People around here are willing to help their neighbors. | PhenX (CoCo) | 2 | 5:1 |
| People in this neighborhood generally don't get along with each other. | PhenX (CoCo) | 2 | 1:5 |
| People in this neighborhood do not share the same values. | PhenX (CoCo) | 2 | 1:5 |
| People in this neighborhood can be trusted. | PhenX (CoCo) | 2 | 5:1 |
| P: I feel safe walking in my neighborhood, day or night | ABCD Parent Neighborhood Safety/Crime Survey Modified from PhenX (NSC) | B / 2 | 1 = Strongly Disagree; 2 = Disagree; 3 = Neutral (neither agree nor disagree); 4 = Agree; 5 = Strongly Agree |
| P: Violence is not a problem in my neighborhood. | PhenX (NSC) | B / 2 | 1:5 |
| P: Neighborhood is safe from crime | PhenX (NSC) | B / 2 | 1:5 |
| **Bullying/ Discrimination** | | | |
| Gets teased a lot | ABCD Parent Child Behavior Checklist Raw Scores Aseba (CBCL) | B / 2 | 0 = Not True; 1 = Somewhat/Sometimes True; 2 = Very True/Often True |
| Problems with bullying at school or in your neighborhood | ABCD (Longitudinal) KSADS-Back | B / 2 | 1 = Yes; 0 = No |
| Ever been cyberbullied * | ABCD Cyber Bully | 2 | 1=Yes; 0=No; 777=Refuse To Answer/NaN |
| Felt discriminated against: because you/family are from another country * | ABCD Youth Discrimination Measure (YDM) | 2 | 1 = Yes; 0 = No; 777 = Don't know/NaN; 999 = Refused to answer/NaN |
| Fel discriminated against because someone thought you were gay, lesbian, or bisexual * | ABCD YDM | 2 | 1 = Yes; 0 = No |
| Felt discriminated against because of race, ethnicity, or color in past year * | ABCD YDM | 2 | 1 = Yes; 0 = No |
| Felt discriminated against because of weight in past year * | ABCD YDM | 2 | 1 = Yes; 0 = No |
| **Mental Health/Temperament** | | | |
| Symptom - History of traumatic event Present | ABCD Parent Diagnostic Interview for DSM-5 Full (KSADS-5) | B / 2 | 0=False, 1=True |
| External CBCL Syndrome Scale (t-score) | ABCD CBCL | B / 2 | continuous |
| Internal CBCL Syndrome Scale (t-score) | ABCD CBCL | B / 2 | continuous |
| Anxiety - all related diagnosis or symptom items | ABCD KSADS-5 | B / 2 | 1=Yes to any question, 0 = No to all questions |
| Depression - all related diagnosis or symptom items | ABCD KSADS-5 | B / 2 | 1=Yes to any question, 0 = No to all questions |
| Anhedonia - symptom past or present (may be related to PTSD) | ABCD KSADS-5 | B / 2 | 1=Yes to any question, 0 = No to all questions |
| Diagnosis - Social Anxiety Disorder (F40.10) past or present | ABCD KSADS-5 | B / 2 | Combined responses: 0=No, 1 = Yes in either Past or Present |
| Symptom - Fear of Social Situations past or present | ABCD KSADS-5 | B / 2 | Combined responses: 0=No, 1=Yes to either past or present |
| Decreased Self Esteem - symptom past or present | ABCD KSADS-5 | B / 2 | 1 = Yes to either question, 0 = No to both |
| Impulsivity - symptom past or present | ABCD KSADS-5 | B / 2 | 1 = Yes to either question, 0 = No to both |
| Impulsive/acts without thinking | ABCD CBCL | B / 2 | 0:2 |
| Would rather be alone than with others | ABCD CBCL | B / 2 | 0:2 |
| Self-conscious or easily embarrassed | ABCD CBCL | B / 2 | 0:2 |
| Withdrawn, doesn't get involved with others | ABCD CBCL | B / 2 | 0:2 |

**Table S2:** *Independent variables selected via shrinkage (Figure 1), for each empathetic outcome, using baseline data (a) and year 2 data (b). All models included sex as a biological variable.*

**(a) Baseline**

| **Considerate of others’ feelings** | **Helpful if someone is hurt, upset, or feeling ill** | **Often offers to help others** | **Does not seem to feel guilty after misbehaving** |
| --- | --- | --- | --- |
| Sex | Sex | Sex | Sex |
| Family often fights | White | Age | Caregiver education |
| Family should come first | BMI | BMI | Population density |
| Share home with relatives | Family often fights | Lives at home full time | US census geographic division  West - Mountain |
| Important to show affection | Family members hit each other | Family often fights | Weekly physical activity |
| Frequency of religious service attendance | Family tries to keep peace | Caregiver warmth | Family rarely openly angry |
| Importance of religious beliefs in youth’s daily life | Parent attempted/committed suicide | Parent attempted/committed suicide | Family members hit each other |
| Gets along with teachers | Family should come first | Family should come first | Parent history of drug issues |
| Has best friend | Parents should teach children to be independent | People should learn to take care of themselves | Children should always do things to make parents happy |
| Externalizing score | Important to have close relationships with extended family | Parents should teach children to be independent | Important to show affection |
| Internalizing score | Parents should be willing to make sacrifices for their children | As children get older they should be allowed to make their own decisions | Importance of religious beliefs in youth’s daily life |
| Fear of social situations | Important to show affection | Important to have close relationships with extended family | Parent English fluency |
| Impulsive (DSM) | Frequency of religious service attendance | Important to show affection | School where students have lots of chances to help decide class activities/rules |
| Impulsive (CBCL) | Importance of religious beliefs in youth’s daily life | Importance of religious beliefs in youth’s daily life | Bullied |
| Would rather be alone | Religion should be an important part of one’s life | Parent English fluency | Externalizing score |
| Self conscious | Parent English fluency | Likes school | Internalizing score |
|  | Youth English fluency | School in person | Anhedonia |
|  | Likes school | Crime is not an issue in neighborhood | Self esteem |
|  | Crime is not an issue in neighborhood | Teased | Impulsive (CBCL) |
|  | Bullied | History of traumatic event | Would rather be alone |
|  | Externalizing score | Externalizing score |  |
|  | Fear of social situations | Internalizing score |  |
|  | Impulsive (DSM) | Fear of social situations |  |
|  | Would rather be alone | Impulsive (DSM) |  |
|  | Self conscious | Impulsive (CBCL) |  |
|  | Withdrawn | Would rather be alone |  |
|  |  | Withdrawn |  |

**(b) Follow-up**

| **Considerate of others’ feelings** | **Helpful if someone is hurt, upset, or feeling ill** | **Often offers to help others** | **Does not seem to feel guilty after misbehaving** | **When angry at someone, says things s/he knows will hurt that person’s feelings** | **Likes taking care of other people** | **Makes fun of how other people Look** | **I left another kid out of an activity or conversation that they wanted to be included in** | **I did not invite a kid to a party or other social event even though I knew the kid wanted to go** |
| --- | --- | --- | --- | --- | --- | --- | --- | --- |
| Sex | Sex | Sex | Sex | Sex | Sex | Sex | Sex | Sex |
| Family size | Age | Family income | US census geographic division  West - Mountain | Caregiver Education | Age | Age | US census geographic division  Northeast - New England | US census geographic region  Northeast |
| US census geographic division  Northeast - Mid-Atlantic | Caregiver education | BMI | BMI | Family size | Family talks about personal problems | US census geographic division  West - Mountain | Family members sometimes get so angry they throw things | Frequency that parents know who child is with |
| US census geographic division  Midwest - East North Central | BMI | Family talks about personal problems | Family rarely openly angry | US census geographic division  Northeast - New England | Family do not share feelings | Sleep duration | Frequency of eating dinner with parents | Important to show affection |
| Number of group sports/activities | Family sense of togetherness | Plenty of time and attention for everyone in family | Family members hit each other | Weekly physical activity | Frequency of youth discussing plans for the coming day with parents | Family members critical of each other | Think of family for important decisions | Parent has strong attachment to ethnic group |
| Family talks about personal problems | Family talks about personal problems | Frequency of youth discussing plans for the coming day with parents | Little group spirit in family | Family members critical of each other | Family gives security | Family members hit each other | Youth religious preference | Gets along with teachers |
| Family gets along | Family cohesion score | Family cohesion score | Family gets along | Family members hit each other | As children get older they should be allowed to make their own decisions | Family often has friends over | Frequency of religious service attendance | Virtual school |
| Parent history of alcohol issues | Family should come first | Family should come first | Parent history of drug issues | Family conflict score | Older siblings role model | Plenty of time and attention for everyone in family | Gets along with teachers | Cyber bullied |
| Family should come first | Important to show affection | Children should be taught that it is their duty to care for parents when they get old | Parent history of depression | When there are problems in life, a person can only count on him or herself | When there are problems in life, a person can only count on him or herself | Family cohesion score | Feels safe at school | Discriminated against because of weight |
| Parents should be willing to make sacrifices for their children | Importance of religious beliefs in youth’s daily life | Family gives security | Parent attempted/committed suicide | Parents should be willing to make sacrifices for their children | Important to show affection | Children should always do things to make parents happy | Finds school boring |  |
| Important to show affection | Parent English fluency | Parents should teach children to be independent | Teased | Finds school boring | Work hard because represent family | People should learn to take care of themselves | Has regular group of kids to hang out with at school/neighborhood |  |
| Importance of religious beliefs in youth’s daily life | School with many chances to be part of classroom discussions/activities | As children get older they should be allowed to make their own decisions | Discrimination for sexual orientation | Externalizing score | Importance of religious beliefs in youth’s daily life | Parents should teach children to be independent | Number of friends |  |
| School where students have lots of chances to help decide class activities/rules | Likes school. | Think of family for important decisions | Externalizing score | Self Esteem | Important to thank God every day | Parents should be willing to make sacrifices for their children | Cyber bullied |  |
| Has best friend | Has best friend | Importance of religious beliefs in youth’s daily life | Internalizing score | Would rather be alone | Important to follow the word of God | Importance of religious beliefs in youth’s daily life | Discrimination for race, ethnicity, skin color |  |
| Teased | Number of friends | Teach children to pray | Anhedonia |  | Gets along with teachers | Parent English fluency | Anxiety |  |
| History of traumatic event | People in this neighborhood do not share the same values | Parent sense of belonging to ethnic group | Impulsive (DSM) |  | Likes school | Likes school | Withdrawn |  |
| Externalizing score | Cyber bullied | Gets along with teachers | Impulsive (CBCL) |  | People in this neighborhood do not share the same values | People in this neighborhood do not share the same values |  |  |
| Internalizing score | Discrimination for race, ethnicity, skin color | Likes school | Withdrawn |  | Discrimination for race, ethnicity, skin color | Cyber bullied |  |  |
| Impulsive (DSM) | Externalizing score | Externalizing score |  |  | Externalizing score | Externalizing score |  |  |
| Impulsive (CBCL) | Internalizing score | Internalizing score |  |  | Internalizing score | Internalizing score |  |  |
| Would rather be alone | Anhedonia | Anhedonia |  |  | Would rather be alone | Social anxiety disorder |  |  |
| Withdrawn | History of traumatic event | History of traumatic event |  |  |  | Withdrawn |  |  |
|  | Fear of social situations | Would rather be alone |  |  |  |  |  |  |
|  | Impulsive (DSM) | Self conscious |  |  |  |  |  |  |
|  | Would rather be alone | Withdrawn |  |  |  |  |  |  |
|  | Withdrawn |  |  |  |  |  |  |  |

**Table S3:** *Responses to selected mental health and temperament variables at baseline and year 2. Note that anxiety and depression values may be an overestimate due to ABCD coding issues. *‘Yes’ to any related questions in the ABCD Parent Diagnostic Interview for DSM-5 Full (KSADS-5).*

|  | | **Baseline** | **Year 2** |
| --- | --- | --- | --- |
|  |  | **n=11062** | **n=9832** |
| **History of Trauma** | **Yes** | 3795 (34.30%) | 3176 (32.30%) |
|  | **No** | 7107 (64.25%) | 6467 (65.78%) |
|  | **Missing** | 160 (1.45%) | 189 (1.92%) |
| **Anxiety*** | **Yes** | 3545 (32.05%) | 2377 (24.18%) |
|  | **No** | 7376 (66.68%) | 7266 (73.90%) |
|  | **Missing** | 141 (1.27%) | 189 (1.92%) |
| **Depression*** | **Yes** | 929 (8.40%) | 915 (9.31%) |
|  | **No** | 9992 (90.33%) | 8728 (88.77%) |
|  | **Missing** | 141 (1.27%) | 189 (1.92%) |
| **Anhedonia*** | **Yes** | 660 (5.97%) | 677 (6.89%) |
|  | **No** | 10261 (92.76%) | 8966 (91.19%) |
|  | **Missing** | 141 (1.27%) | 189 (1.92%) |
| **Social Anxiety Disorder (past or present)** | **Yes** | 430 (3.89%) | 370 (3.76%) |
|  | **No** | 10491 (94.84%) | 9273 (94.32%) |
|  | **Missing** | 141 (1.27%) | 189 (1.92%) |
| **Fear of Social Situations (past or present)** | **Yes** | 1075 (9.72%) | 836 (8.50%) |
|  | **No** | 9846 (89.01%) | 8807 (89.58%) |
|  | **Missing** | 141 (1.27%) | 189 (1.92%) |
| **Self Esteem (DSM)** | **Yes** | 407 (3.68%) | 424 (4.31%) |
|  | **No** | 10507 (94.98%) | 9219 (93.77%) |
|  | **Missing** | 148 (1.34%) | 189 (1.92%) |
| **Impulsive (DSM)** | **Yes** | 3077 (27.81%) | 1113 (11.32%) |
|  | **No** | 7837 (70.85%) | 8530 (86.76%) |
|  | **Missing** | 148 (1.34%) | 189 (1.92%) |
| **Impulsive or acts without thinking (CBCL)** | **Not True** | 8568 (77.46%) | 7841 (79.75%) |
|  | **Somewhat /Sometimes True** | 2153 (19.46%) | 1701 (17.30%) |
|  | **Very/Often True** | 334 (3.02%) | 235 (2.39%) |
|  | **Missing** | 7 (0.06%) | 55 (0.56%) |
| **Would rather be alone than with others** | **Not True** | 9414 (85.11%) | 7871 (80.06%) |
|  | **Somewhat True** | 1508 (13.63%) | 1721 (17.50%) |
|  | **Very True** | 133 (1.20%) | 185 (1.88%) |
|  | **Missing** | 7 (0.06%) | 55 (0.56%) |
| **Self-conscious or Easily Embarrassed** | **Not True** | 6452 (58.33%) | 5862 (59.62%) |
|  | **Somewhat True** | 3992 (36.09%) | 3416 (34.74%) |
|  | **Very True** | 611 (5.52%) | 499 (5.08%) |
|  | **Missing** | 7 (0.06%) | 55 (0.56%) |

**Table S4:** *Baseline and follow-up model statistics for: (a) Associations between anxiety/depression and empathetic behaviors; Part 2: Associations between externalizing/internalizing score and empathetic behaviors; Part 3: Associations between anxiety/depression and internalizing/externalizing behaviors; Part 4: Associations between anxiety/depression + internalizing/externalizing behaviors and empathetic behaviors. Cells are empty where the association is nonsignificant or where the model was invalid.*

|  | **Regression Coefficient** | **Standard Error (SE)** | **P-value** | **Wald Statistic** | **Regression Coefficient** | **Standard Error (SE)** | **P-value** | **Wald Statistic** |  | |  |  |
| --- | --- | --- | --- | --- | --- | --- | --- | --- | --- | --- | --- | --- |
|  | **Baseline (n=11062)** | | | | **Year 2 (n=9832)** | | | | |  | | |
|  | | | | | | | | |  | |  |  |
| **Anxiety → Empathetic/CU Behaviors** | | | | | | | | | |  | | |
| Considerate of other people's feelings | -0.073 | 0.010 | <0.001 | 53.294 | -0.074 | 0.012 | <0.001 | 40.502 |  | |  |  |
| Helpful if someone is hurt, upset, or feeling ill | -0.052 | 0.009 | <0.001 | 32.875 | -0.056 | 0.011 | <0.001 | 28.320 |  | |  |  |
| Often offers to help others (parents, teachers, other children) | -0.066 | 0.011 | <0.001 | 37.006 | -0.123 | 0.013 | <0.001 | 88.669 |  | |  |  |
| Doesn't seem to feel guilty after misbehaving | 0.084 | 0.008 | <0.001 | 113.954 |  |  |  |  |  | |  |  |
| When angry at someone says things s/he knows will hurt that person's feelings | Not available at baseline | | | | 0.238 | 0.027 | <0.001 | 80.682 |  | |  |  |
| Likes taking care of other people |  |  |  |  | -0.144 | 0.024 | <0.001 | 36.211 |  | |  |  |
|  | | | | | | | | |  | |  |  |
| **Depression → Empathetic/CU Behaviors** | | | | | | | | |  | |  |  |
| Considerate of other people's feelings | -0.117 | 0.017 | <0.001 | 45.949 | -0.138 | 0.018 | <0.001 | 62.197 |  | |  |  |
| Helpful if someone is hurt, upset, or feeling ill | -0.067 | 0.016 | <0.001 | 18.716 | -0.079 | 0.016 | <0.001 | 25.510 |  | |  |  |
| Often offers to help others (parents, teachers, other children) | -0.081 | 0.019 | <0.001 | 18.947 | -0.132 | 0.020 | <0.001 | 45.808 |  | |  |  |
| Doesn't seem to feel guilty after misbehaving | 0.125 | 0.014 | <0.001 | 85.785 |  |  |  |  |  | |  |  |
| When angry at someone says things s/he knows will hurt that person's feelings | Not available at baseline | | | | 0.312 | 0.040 | <0.001 | 61.534 |  | |  |  |
| Likes taking care of other people |  |  |  |  | -0.209 | 0.036 | <0.001 | 34.094 |  | |  |  |
| Makes fun of how other people look |  |  |  |  | 0.138 | 0.030 | <0.001 | 20.899 |  | |  |  |
|  | | | | | | | | |  | |  |  |
| **Anxiety → Externalizing/Internalizing Behavior Scores** | | | | | | | | |  | |  |  |
| Externalizing | 4.872 | 0.201 | <0.001 | 589.451 | 3.857 | 0.253 | <0.001 | 232.568 |  | |  |  |
| Internalizing | 8.072 | 0.201 | <0.001 | 1616.703 | 8.674 | 0.257 | <0.001 | 1140.893 |  | |  |  |
| **Depression → Externalizing/Internalizing Behavior Scores** | | | | | | | | | |  | | |
| Externalizing | 6.624 | 0.349 | <0.001 | 359.913 | 5.345 | 0.373 | <0.001 | 205.062 |  | |  |  |
| Internalizing | 8.360 | 0.363 | <0.001 | 529.752 | 8.160 | 0.397 | <0.001 | 423.598 |  | |  |  |
|  | | | | | | | | |  | |  |  |
| **Anxiety + Externalizing Behaviors → Empathetic/CU Behaviors** | | | | | | | | | |  | | |
| Often offers to help others (parents, teachers, other children) |  |  |  |  | -0.059 | 0.014 | <0.001 | 17.005 |  | |  |  |
| Makes fun of how other people look | Not available at baseline | | | | -0.079 | 0.022 | <0.001 | 13.130 |  | |  |  |
| **Anxiety + Internalizing Behaviors → Empathetic/CU Behaviors/Emotions** | | | | | | | | | |  | | |
| Often offers to help others (parents, teachers, other children) |  |  |  |  | -0.050 | 0.016 | 0.001 | 10.306 |  | |  |  |
| **Depression + Externalizing Behaviors → Empathetic/CU Behaviors** | | | | | | | | | |  | | |
| Often offers to help others (parents, teachers, other children) |  |  |  |  | -0.061 | 0.021 | 0.004 | 8.224 |  | |  |  |
| **Depression + Internalizing Behaviors → Empathetic/CU Behaviors** | | | | | | | | | |  | | |
| Considerate of other people's feelings | -0.063 | 0.018 | <0.001 | 12.882 | -0.079 | 0.020 | <0.001 | 16.102 |  | |  |  |
| Often offers to help others (parents, teachers, other children) |  |  |  |  | -0.079 | 0.022 | <0.001 | 12.987 |  | |  |  |
| Doesn't seem to feel guilty after misbehaving |  |  |  |  | 0.057 | 0.015 | <0.001 | 14.052 |  | |  |  |
| When angry at someone says things s/he knows will hurt that person's feelings | Not available at baseline | | | | 0.126 | 0.045 | 0.005 | 7.913 |  | |  |  |
| Likes taking care of others |  |  |  |  | -0.134 | 0.040 | <0.001 | 11.170 |  | |  |  |
